# Targeted editing of the *PSIP1* gene encoding LEDGF/p75 protects cells against HIV infection

**DOI:** 10.1038/s41598-019-38718-0

**Published:** 2019-02-20

**Authors:** Yulia Lampi, Dominique Van Looveren, Lenard S. Vranckx, Irina Thiry, Simon Bornschein, Zeger Debyser, Rik Gijsbers

**Affiliations:** 1 Laboratory for Viral Vector Technology and Gene Therapy, Department of Pharmaceutical and Pharmacological Sciences, KU Leuven, 3000 Leuven, Belgium; 2 Laboratory for Molecular Virology and Drug Discovery, Department of Pharmaceutical and Pharmacological Sciences, KU Leuven, 3000 Leuven, Belgium; 3 Leuven Viral Vector Core, KU Leuven, 3000 Leuven, Belgium; 4 Center for Human Genetics, VIB Center for the Biology of Disease, KU Leuven, 3000 Leuven, Belgium

## Abstract

To fulfill a productive infection cycle the human immunodeficiency virus (HIV) relies on host-cell factors. Interference with these co-factors holds great promise in protecting cells against HIV infection. LEDGF/p75, encoded by the *PSIP1* gene, is used by the integrase (IN) protein in the pre-integration complex of HIV to bind host-cell chromatin facilitating proviral integration. LEDGF/p75 depletion results in defective HIV replication. However, as part of its cellular function LEDGF/p75 tethers cellular proteins to the host-cell genome. We used site-specific editing of the *PSIP1* locus using CRISPR/Cas to target the aspartic acid residue in position 366 and mutated it to asparagine (D366N) to disrupt the interaction with HIV IN but retain LEDGF/p75 cellular function. The resulting cell lines demonstrated successful disruption of the LEDGF/p75 HIV-IN interface without affecting interaction with cellular binding partners. In line with LEDGF/p75 depleted cells, D366N cells did not support HIV replication, in part due to decreased integration efficiency. In addition, we confirm the remaining integrated provirus is more silent. Taken together, these results support the potential of site-directed CRISPR/Cas9 mediated knock-in to render cells more resistant to HIV infection and provides an additional strategy to protect patient-derived T-cells against HIV-1 infection as part of cell-based therapy.

## Introduction

Acquired immunodeficiency syndrome (AIDS) is a life-threatening acquired disorder resulting from an infection with the human immunodeficiency virus (HIV) and the subsequent progressive loss of CD4^+^ T cells^1^. Over the years, HIV research has identified several druggable targets, resulting in potent drugs that have considerably improved survival and long-term clinical management of HIV-infected individuals. The advent of combination antiretroviral therapy (cART) allowed HIV replication to be suppressed to below detection level^2^. However, even with strict adherence to the therapeutic regimen, patients remain chronically infected since cART is unable to clear latent viral reservoirs and thus necessitate lifelong treatment^3,4^. Efficacy of the regimen is strongly determined by the degree of compliance, but inevitably comes with a substantial financial cost and drug-related adverse effects such as drug-resistant escape mutants, cumulative toxicities, persistent immune dysfunction and accelerated aging phenomena. Hence, persistent viral reservoirs represent the main barrier towards a cure for HIV.

Diminishing the latent reservoir and/or preventing *de novo* infection events are potential mechanisms by which a cure can be accomplished. To date HIV virus has only been eradicated in a single person, the ‘Berlin patient’^5^. In this case, cure was achieved following allogeneic hematopoietic stem cell (HSC) transplantation from a donor homozygous for *CCR5Δ*32, a natural CCR5 mutant that prevents entry of HIV-1 (R5 strains). The latter success has fueled interest to develop alternative treatment strategies and engineer patient-derived immune cells that lack host factors that are essential for HIV replication and pathogenesis to achieve a permanent cure^6,7,8^. This sole case of HIV cure underscores gene therapy as a promising route to cure HIV^9–11^. A wide variety of anti-HIV strategies has been tested in cell-culture and animal models, and some of them have progressed to clinical trials^12^. Zinc finger nuclease (ZFN)-mediated genomic disruption in autologous CD4^+^ T-cells of the CCR5 coding region resulted in efficient inhibition of HIV-1 infection^13^. In line, more recently a CRISPR/Cas9 array platform was developed to ablate multiple HIV-1 host factors simultaneously, generating primary human T-cells refractory to HIV infection, underscoring the potential of genome editing for therapeutic application against HIV-1 infection^14^. An important consideration that emerged from the study was the notion of deleterious consequences of complete gene ablation *in vivo*^15^. A problem that can be resolved by engineering specific modifications within the HIV co-factors to disrupt the interaction with the virus while retaining their normal cellular function.

**Figure 1:**
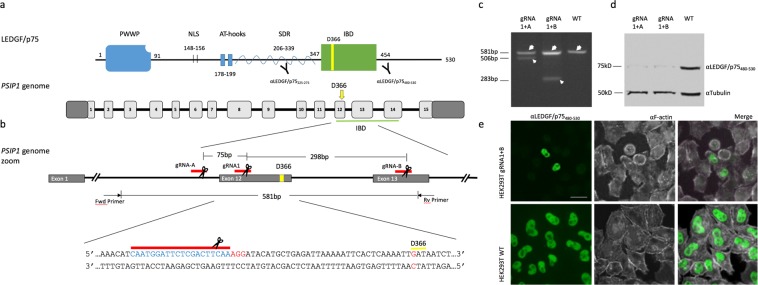
Guide RNA adjacent to the coding sequence D366 shows efficient disruption of the *PSIP1* gene. (**a**) Schematic representation of LEDGF/p75 protein with indication of the epitope sites of respective antibodies used in Western analysis. Below the human *PSIP1* locus on chromosome 9 is depicted showing the different exons as light grey boxes. IBD is underlined in green. (**b**) Schematic of representing the location of the different gRNA that were used (red lines), gRNA1 close to D366 and two additional supporting gRNAs (gRNA_A, gRNA_B). D366 is shown in yellow. The expected PCR fragment sizes are indicated as well as the predicted deletions for the different gRNA combinations. Below the targeted gDNA sequence is shown. D366 is boxed in green, the PAM site is shown in red and the landing site of gRNA1 is shown in blue. (**c**) Agarose gel analysis showing truncated amplicons generated by DNA cleavage guided by a pair of gRNAs. Genomic DNA was extracted from polyclonal cell populations and PCR amplified using Fwd and Rv primers indicated in panel (**b**). The WT amplicon is indicated by the large arrow head. The lower migrating bands (small arrow head) indicate segmental deletion. (**d**) Western blot analysis showing LEDGF protein in a polyclonal HEK293T population transfected with the indicated gRNA pairs. Wild-type 293T cells (WT) are shown as control. (**e**) Immunocytochemical staining of endogenous LEDGF showing nuclear localization in WT and CRISPRed polyclonal HEK239T cells. Phalloidin-stained F-actin in white is shown as a counterstain. The respective antibodies used are indicated above. Scale Bar: 10 μm.

A crucial step in the viral replication cycle is the efficient and stable integration within the host cell genome, which is catalyzed by the viral integrase (IN) enzyme through the direct interaction with the host cell protein Lens Epithelial Derived Growth Factor p75 (LEDGF/p75), encoded by the *PSIP1* gene on chromosome 9. LEDGF/p75 is used as cofactor by all lentiviruses to tether the viral pre-integration complex (PIC) to the host chromatin^16–18^, thus guiding the integration toward actively-transcribed regions of the genome^19,20^. LEDGF/p75 is also an epigenetic reader consisting of an assembly of conserved chromatin interacting domains at the N-terminus and a protein binding C-terminus (Fig. 1a). The N-terminal end consists of PWWP (Proline-Tryptophan-Tryptophan-Proline) domain responsible for recognition of methylated histone tails^21^, a nuclear localization signal (NLS)^22^, two AT hook-like motifs and three relatively charged regions (CR)^23^. In the C-terminal region, the integrase (IN) binding domain (IBD; aa347–429) functions as a protein hub, which interacts with several cellular proteins and protein complexes, as well as the lentiviral IN (Fig. 1a)^22,24,25^. A shorter protein isoform resulting from alternative splicing, LEDGF/p52, shares the N-terminal portion of the protein, but lacks the IBD and is not implicated in lentiviral replication.

LEDGF/p75 has been validated as a potential target to render primary cells resistant to HIV infection in a humanized mouse model for HIV infection^25^. Both LEDGF/p75 knock-out and expression of the IBD prevented HIV infection of primary human T-cells^14,25^. However, since the IBD also recruits cellular binding partners and complexes to the chromatin a more subtle approach is preferred that exclusively targets the interaction with the HIV IN, leaving the interactions with other cellular binding partner unaffected^24,26–33^. Development of small molecule inhibitors that bind the IN dimer cleft (LEDGINs) and interfere with the interaction between LEDGF/p75 and IN resulted in potent inhibition of HIV replication^34^. NMR experiments revealed an almost identical interaction interface for all cellular binding partners of LEDGF/p75, consisting of an intrinsically unstructured motif, coined IBD interaction motif (IBM)^24,35^. Contrary to the cellular binding partners, the LEDGF/p75 binding site on HIV IN is structurally prearranged and covers a much larger hydrophobic surface area when binding LEDGF/p75^24,35^. LEDGF/p75 binds the HIV IN dimer via the protruding aspartic acid residue at position 366 (D366)^36,37^. Mutation of the latter residue into alanine or asparagine abrogates IN binding without affecting the binding of the endogenous binding partners of LEDGF/p75^28–30^.

Here, we demonstrate that a precise, single-point mutation of LEDGF D366 at the genomic level into asparagine using CRISPR/Cas9 technology protects cells against productive HIV infection, leaving the interaction with endogenous proteins unaffected.

## Results

### Design and validation of a human *PSIP1*-specific gRNA adjacent to the D366 codon

LEDGF/p75 acts as a protein hub, and thus therapeutic ablation may affect several endogenous pathways that are regulated through LEDGF/p75^14,28–32,38^. LEDGF/p75 binds the HIV IN dimer interface via the aspartic acid residue at position 366 located in the IBD^36,37^. Well-characterized single amino-acid substitutions of the D366 in the IBD have been described that specifically disrupt IN binding (D366A/N)^36^, leaving the interaction with other cellular binding partners of LEDGF/p75 unaffected^24,29,39,40^. Therefore, we opted to engineer the specific mutation of the aspartic residue 366 (genomically encoded in exon 12 of *PSIP1* gene) to asparagine (N)^36,37^ using CRISPR/Cas9 technology with a DNA oligo template for homology directed repair (Fig. 1a). To that end, a CRISPR/Cas9 guide RNA (gRNA) was designed to target a site 39 bp upstream of the D366 codon, designated gRNA1 (Fig. 1b and Supplemental Table S1). In addition, two supplementary guide RNAs were designed to recognize regions upstream and downstream of the desired site to evaluate gRNA1-mediated cutting efficiency (intronically upstream of exon 12 and within exon 13, referred to as gRNA-A and -B, respectively; Fig. 1b, Supplemental Table S1). CRISPR/Cas9 introduces DNA double-stranded breaks (DSB) that are typically repaired by non-homologous end-joining (NHEJ). In a first step, HEK293T cells were transfected with a pair of CRISPR/Cas plasmids encoding gRNA1-A and gRNA1-B, respectively. Theoretically, this method results in single cuts for either guide but occasionally a simultaneous cut generates a segmental deletion that is detectable following PCR amplification of the genomic DNA. Primers were designed such that wild-type *PSIP1* yielded an amplicon of 581 bp (large arrow heads; Fig. 1c). PCR analysis showed a prominent band at the expected 581 bp size for the respective polyclonal cell populations. In addition, we observed truncated amplicons of 506 bp when combining the gRNA1 and gRNA-A pair and a 283 bp amplicon for gRNA1 and gRNA-B combination (small arrow heads; Fig. 1c), corresponding to the calculated fragments, validating the individual gRNAs. *PSIP1* disruption at the genomic level in the polyclonal *PSIP1*-deleted HEK293T cell population was further demonstrated by a marked drop in LEDGF/p75 protein levels using an antibody that binds the C-terminal end of LEDGF/p75 (αLEDGF_480–530_; Fig. 1d). Note that some residual LEDGF/p75 protein is still detectable, as expected for a polyclonal population where *PSIP1* deletion is limited by transfection efficiency. In line, immunocytochemistry corroborated loss of nuclear LEDGF/p75 in the large majority of cultured cells (94%; Fig. 1e). Together these data demonstrate that gRNA1 is functional and thus can be used in combination with a DNA oligo template for homology directed repair (HDR) to generate D366N.

### Characterization of gRNA1 LEDGF/p75 knock-out in HEK293T cells

We anticipated that the frequency of HDR events resulting in a homozygous D366N clone would be low, and that the most likely case would be obtaining heterozygous D366N mutants where the second allele contained a *PSIP1*-disrupting indel. Therefore, it was important to examine the phenotype of *PSIP1* KO cells.

**Figure 2:**
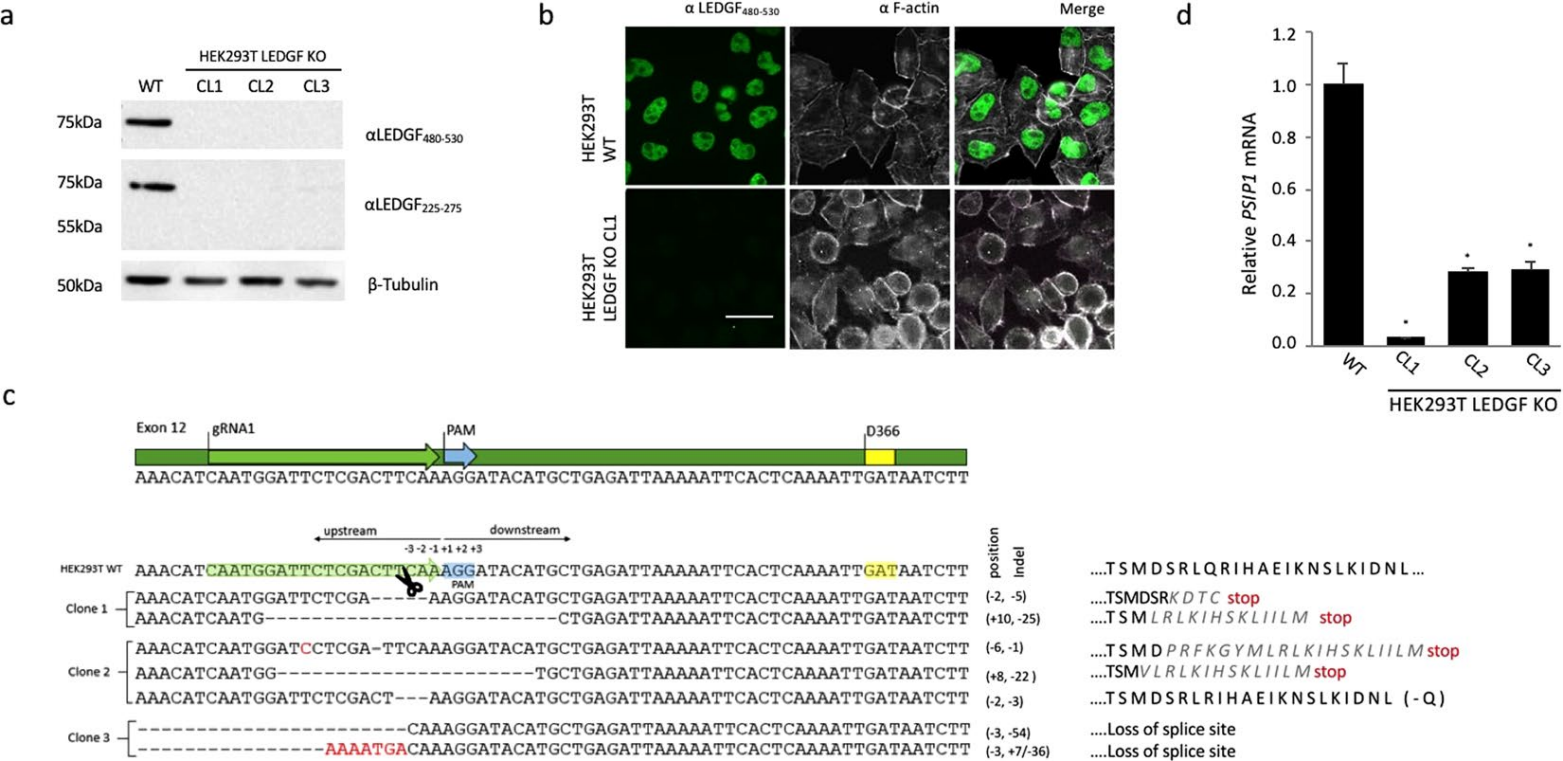
Characterization of HEK293T_LEDGF KO cell lines using a single gRNA1. (**a**) Western blot analysis of LEDGF/p75 protein in 3 LEDGF KO clones. (**b**) Immunocytochemical analysis of wild-type HEK293T (WT) cells and monoclonal LEDGF KO cells showing nuclear localization of LEDGF/p75 as dense, fine speckles in WT cells (shown in green) and the lack thereof in the KO cell line. Phalloidin (white) was used to counterstain cytoplasmic F-actin. LEDGF KO (clone1) image is shown and is representative of all 3 isolated KO cell lines. (**c**) Sanger sequencing of the indel profile generated in the region targeted by gRNA1 (“−” indicates deletion, substitutions indicated in red) and the predicted changes in the amino acid sequence of exon 12 on the right. (**d**) *PSIP1* mRNA expression levels with standard deviation relative to β-actin in the respective knock-out clones as determined by qPCR. Error bars represent SD. Student’s *t* test was performed using GraphPad Prism 7.0 software. Sample means were considered significantly different from the WT control at p < 0.05 (*). Scale Bar: 10 µm.

We used transfection to introduce gRNA1 and Cas9 into HEK293T cells. Monoclonal LEDGF/p75 KO cell lines were isolated via limiting dilution. Individual clones were validated for *PSIP1* disruption by Western analysis and immunocytochemistry (clone 1, 2 and 3, respectively; Fig. 2a,b). Western blot showed undetectable levels of LEDGF/p75 protein (αLEDGF_480–530_, Fig. 2a). In line, no detectable LEDGF/p75 protein was present in immunocytochemistry (clone 1; Fig. 2b). Next, we analyzed the gRNA1 target site for the clones lacking LEDGF/p75 protein in more detail. Genomic DNA regions of *PSIP1* surrounding the PAM site were PCR amplified and subcloned for DNA sequencing (Fig. 2c), showing insertions, deletions or mutations (indels) in close proximity to the PAM site (ranging from 2 to 54 bp) (Fig. 2c). In most alleles, deletions caused a shift in the open reading frame, leading to a premature translation stop (clone 1; Fig. 2c). In HEK293T_LEDGF KO clone 3, 54 bp and 36 bp were deleted in allele 1 and allele 2, respectively, resulting in elimination of the intron/exon boundary at the start of exon 12 (Fig. 2c). In HEK293T_LEDGF KO clone 2, the targeted resequencing revealed the presence of 3 distinct alleles with variable indels (Supplemental Fig. 1). It has previously been observed that HEK293T cell line may exhibit variable hypotriploidy, however, triploidy has never been reported for chromosome 9^41^.

Even though genomic analysis predicted the potential expression of a truncated form of LEDGF/p75, we were unable to detect these by Western analysis when using an antibody that recognizes an epitope upstream of the IBD domain (αLEDGF_225–275_; Fig. 2a). We further analyzed *PSIP1* gene transcription by performing qPCR (Fig. 2d). *PSIP1* mRNA expression was significantly reduced in all three KO lines (96%, 71% and 70% depletion in clone 1, 2 and 3, respectively; *p* < 0.001 Student’s *t* test compared to WT), suggesting that the disruption of the genomic *PSIP1* sequence influences protein expression levels by affecting mRNA stability.

### Generation of *PSIP1* D366N mutation at gDNA level in HEK293T cell line

**Figure 3:**
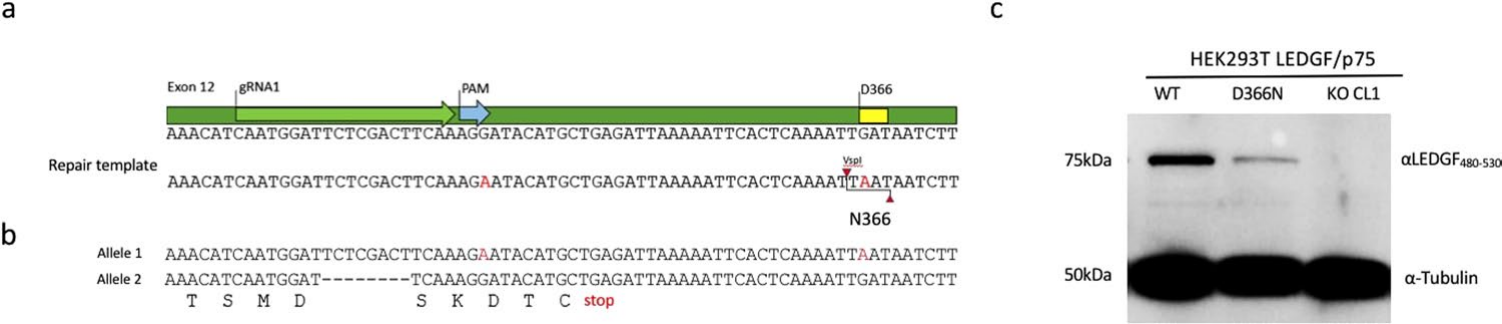
HDR template design and screening. (**a**) Schematic representation of exon12 sequence of *PSIP1 gene* harboring D366 residue. Below the designed HDR template as a ssDNA oligo is shown with red characters indicating the sites of nucleotide substitutions, generating a *VspI* restriction site at D366N and deleting the PAM site. (**b**) Sanger sequencing results for the HEK293T_LEDGF D366N clone that was identified, showing consensus between HDR template and allele 1 (substitution indicated in red). The sequence for allele 2 shows a 8-nucleotide deletion (“−” indicates deletion) and the translation (capital letters below) resulting in a premature stop codon (underlined). (**c**) Western blot analysis showing the LEDGF/p75 levels in D366N clone compared to WT levels.

A 98 bp single-stranded DNA oligo template for HDR (Supplemental Table S1) was designed to contain a point mutation converting D366 into N (GAT to AAT) (Fig. 3a). Introduction of the point mutation in the repair template created a *Vsp*I restriction site (A^TTAAT), which was used in the screening process to identify single cell clones with the D336N mutation. An additional silent nucleotide substitution was added to eliminate the PAM site (AGG to AGA), preventing continuous cutting by the CRISPR/Cas9 complex (Fig. 3a). HEK293T cells were transfected with the CRISPR/Cas9 plasmid carrying gRNA1 targeting *PSIP1* exon12 along with the HDR template in a form of a single-stranded DNA oligo. Clones were screened by immunocytochemistry to identify the LEDGF KO clones. LEDGF/p75-positive clones were subjected to PCR amplification of the genomic DNA to identify D366N mutants: the amplicon for the wild-type or a LEDGF KO allele would give rise to a 581 bp fragment, whereas amplicons carrying D366N yielded two fragments following digestion with *Vsp*I at 296 bp and 285 bp (Supplemental Fig. 2). For a single allelic change, the wild-type band at 581 bp would also be present. A total of 38 individual clones were screened. Two clones were identified based on the predicted PCR banding pattern (clone 4 and 35, respectively; Supplemental Fig. 2) and were analysed in detail by subcloning of the individual isolated amplicons and subsequent sequencing of each allele. Clone 4 contained the desired point mutation at D366 residue in one of the alleles but unfortunately also had indels in the sequence upstream of D366N (Supplemental Fig. 3). In clone 35, one of the alleles matched the D366N HDR template and the other allele contained an 8-nucleotide deletion at the DSB site resulting in a LEDGF KO genotype (Fig. 3b). The sequencing result of clone 35 was corroborated by Western blot analysis showing the LEDGF D366N protein with the same gel migration as the wild-type protein, whereas no protein was detected for the control cell line, LEDGF KO clone1 (Fig. 3c).

### LEDGF D366N mutant fails to interact with HIV integrase in HEK293T

**Figure 4:**
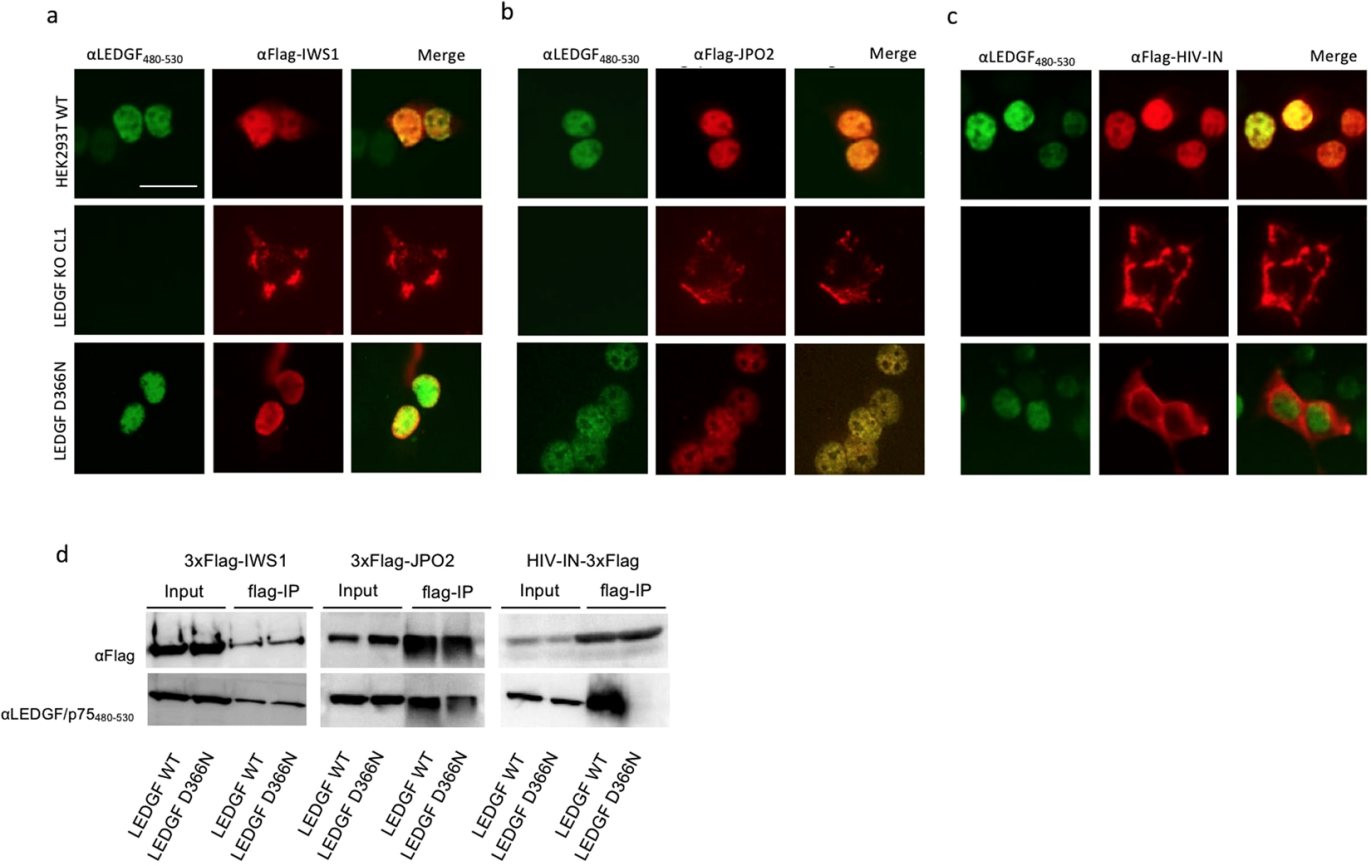
LEDGF D366N mutant is uncoupled from HIV-IN but interacts with other binding partners. (**a**–**c**) *In vivo* co-localization of the LEDGF D366N and cellular binding partners. Wild-type HEK293T, LEDGF KO and LEDGF D366N cells were transfected with flag-tagged IWS1, JPO2 and HIV-IN (panels a, b and c, respectively) and fixed 30 hrs later. Fluorescence microscopy to assess localization of proteins: LEDGF/p75 and LEDGF D366N were detected using αLEDGF_480–530_ (shown in green), whereas the transfected flag-tagged proteins were detected with αFlag Ab (shown in red). (**a**,**b**) LEDGF WT (top panel) and LEDGF D366N (lower panel) co-localizes with IWS1 and JPO2. (**c**) LEDGF D366N mutant did not co-localize with HIV-IN (lower panel), while in HEK293T WT cells HIV-IN is retained in the nucleus by binding to LEDGF/p75 (top panel). Scale Bar: 10 µm. (**d**) Co-immunoprecipitation of LEDGF/p75 and LEDGF D366N by cellular binding partners. HEK293T_LEDGF KO cells were transfected with either WT or D366N mutant LEDGF plasmid along with either one of the plasmids encoding Flag-tagged binding partners: IWS1, JPO2 or HIV IN. Cells were lysed 24 h later and lysates were incubated with anti-FLAG® M2-agarose affinity resin to capture the binding partner protein, which was then visualized by Western blot using αFlag Ab and αLEDGF_480–530_.

Mutation of D366 into N only affects the interaction of LEDGF/p75 with lentiviral IN^36^, leaving the interaction with cellular binding partners unaffected^28–30^. Therefore, we evaluated the capacity of the genomic LEDGF D366N mutant to interact with the known cellular interacting partners JPO2 and IWS1^24,29,40^, whose binding sites are mutually exclusive from HIV-IN but lie in close proximity to the D366 residue. Endogenous LEDGF/p75 and LEDGF D366N were readily detected in HEK293T cells using α-LEDGF_480–530_ (Fig. 4) and revealed the typical LEDGF dense fine speckled pattern in the nucleus (Fig. 4; green fluorescence, left column of a, b and c). The nuclear distribution of LEDGF/p75 (HEK293T_WT) and LEDGF D366N was comparable, suggesting that D366N mutation did not affect the subcellular localization of LEDGF/p75. Next, these cells, together with LEDGF KO cells as control, were transiently transfected with plasmids encoding flag-tagged versions of IWS1, JPO2 and HIV-IN, respectively, to assess co-localization (α-flag, red fluorescent signal, Fig. 4). In both HEK293T_WT and HEK293T_LEDGF D366N co-localized with IWS1 and JPO2 in the nucleus, indicating that the D366N mutation did not affect the interaction with these proteins (right column, lower panel; Fig. 4a,b). Transiently expressed HIV-IN located to the nucleus in HEK293T WT cells, whereas it distributed throughout the cell for HEK293T_LEDGF D366N with a preference for the cytoplasm, suggesting it failed to bind LEDGF D366N, in line with HEK293T_KO clone 1 (Fig. 4c). To further confirm that the interaction between LEDGF D366N and HIV IN was disrupted but was retained with JPO2 and IWS1, we performed coIP experiments by transiently expressing the LEDGF WT or mutant D366N along with the respective binding partners in LEDGF KO HEK293T cells. As seen in co-localization experiment above, coIP corroborated the interaction between LEDGF D366N and JPO2 or IWS1 (Fig. 4d, left and middle panels) to be similar to the WT condition. However, HIV IN did not coIP LEDGF D366N (Fig. 4d, right panel), whereas the LEDGF WT protein was precipitated.

Together, these experiments validate our approach and demonstrate that mutation of the protruding aspartic acid residue D366 of LEDGF/p75 disrupts binding of HIV IN, while maintaining the interaction with cellular binding partners.

### CRISPR-based *PSIP1* knock-out renders SupT1 cells resistant to HIV-1 infection

**Figure 5:**
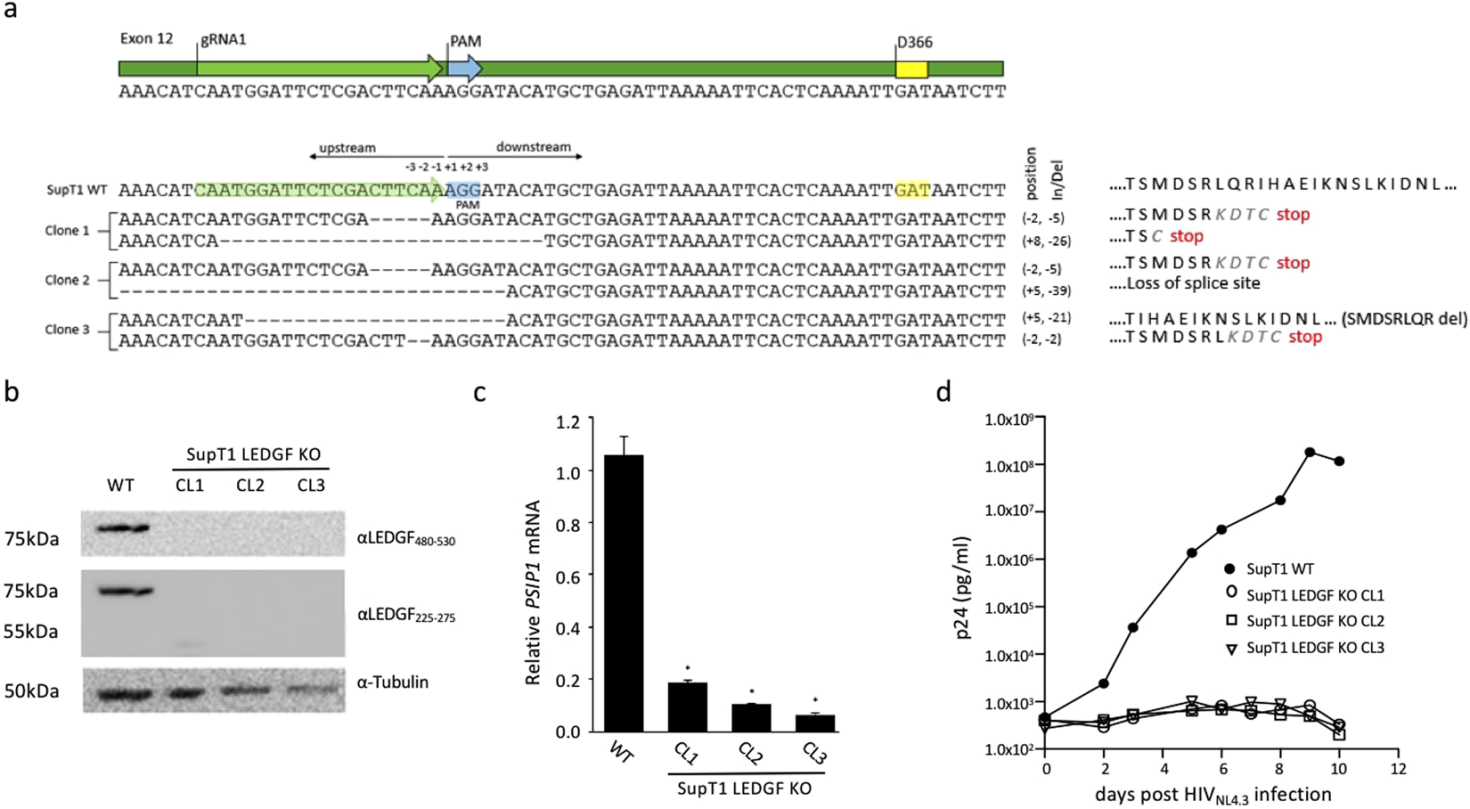
HIV replication is severely affected in SupT1_LEDGF KO cells. (**a**) Schematic representation of a detailed zoom of exon12 sequence of the *PSIP1* gene harboring D366. Sanger sequencing of the indel profile generated in the region targeted by gRNA1 (“−” indicates deletion, substitution indicated in red) and the predicted changes in the amino acid sequence of exon 12 shown on the right. (**b**) Western analysis of LEDGF/p75 protein in the 3 isolated LEDGF KO cell lines. (**c**) *PSIP1* mRNA expression relative to β-actin in the respective SupT1_LEDGF KO clones as determined by qPCR. Error bars represent SD. Student’s *t* test was performed using GraphPad Prism 7.0 software. Sample means were considered significantly different from the WT control at p < 0.05 (*). (**d**) HIV-1 replication assay. The respective cell lines were challenged with the laboratory HIV_NL4.3_ strain at a final concentration of 5.0*10^2^ pg p24. Viral replication was monitored by daily sampling of p24 in the cell culture supernatant. The graph shows a representative infection experiment out of three independent trials.

Human CD4^+^ T cells are the major target cells of HIV-1 infection, and thus most interesting to render HIV resistant. In a first step, we set out to determine whether CRISPR/Cas9-mediated *PSIP1* gene disruption protects SupT1 cells from HIV-1 infection. In order to efficiently disrupt *PSIP1* in a polyclonal population, we generated SupT1 cells constitutively expressing Cas9 nuclease. The SupT1-Cas9 cells were then electroporated with a plasmid encoding the gRNA1 and GFP. GFP-positive cells were sorted to enrich the percentage of *PSIP1*-deleted cells. The resulting polyclonal populations were FACS-analyzed to assess LEDGF/p75 expression levels (Supplemental Fig. 4a,b). Non-sorted cells displayed a 72% knock-out efficiency, whereas GFP-sorted cells showed an 88% knock-out efficiency using gRNA1. Individual LEDGF KO clones were obtained through limiting dilution and screened by immunocytochemistry (using α-LEDGF_480–530_; not shown). For three of the monoclonal cell lines (SupT1_LEDGF KO clone 1, 2 and 3, respectively), we amplified and sequenced the genomic region flanking the PAM cleavage site to determine the indel profile for both alleles (Fig. 5a). The indels in SupT1_LEDGF KO clone 1 and clone 2 generated a frameshift mutation in both alleles, resulting in a premature stop codon (Fig. 5a). One of the alleles in clone 2 had a deletion beyond the intron11-exon12 boundary. In SupT1_LEDGF KO clone 3, the second allele had an 8-amino-acid deletion but remained in frame leaving the possibility of a truncated form of LEDGF/p75. Loss of LEDGF/p75 protein was corroborated by Western analysis using the C-terminal specific antibody (α-LEDGF_480–530_) as well as the antibody recognizing the epitope in the CR region upstream of IBD (α-LEDGF_225–275_), to identify a potentially truncated form of LEDGF/p75 (Fig. 5b). Whereas both antibodies readily detected LEDGF/p75 in wild-type SupT1 cells (SupT1_WT), no detectable protein was observed for any of the clones tested, implying that the indels generated at the site of cleavage destabilized the protein and/or the *PSIP1* mRNA. In line with the analysis completed on HEK293T_LEDGF KO cell lines, we assessed *PSIP1* mRNA levels and showed significant reduction in all three SupT1_LEDGF KO lines (Fig. 5c; 83%, 90% and 93% depletion, respectively), again demonstrating that disruption of *PSIP1* at genomic level results in substantially lower mRNA levels and in loss of LEDGF/p75 protein.

Next, we assessed whether the SupT1_LEDGF KO clones supported HIV-1 replication and challenged the respective cell lines with the laboratory strain HIV_NL4.3_. In a viral replication assay SupT1_WT cells consistently showed peak viral replication between day 4 and 7 post infection (Fig. 5d), whereas in the respective knock-out cell lines HIV failed to replicate when followed for 10 days post infection. Thus, CRISPR/Cas9 mediated disruption of *PSIP1* in SupT1 cells is able to prevent HIV replication, in line with previous work^14,26^.

### Residual HIV-1 integrants adopt a latent phenotype in *PSIP1* knock-out SupT1 cells

**Figure 6:**
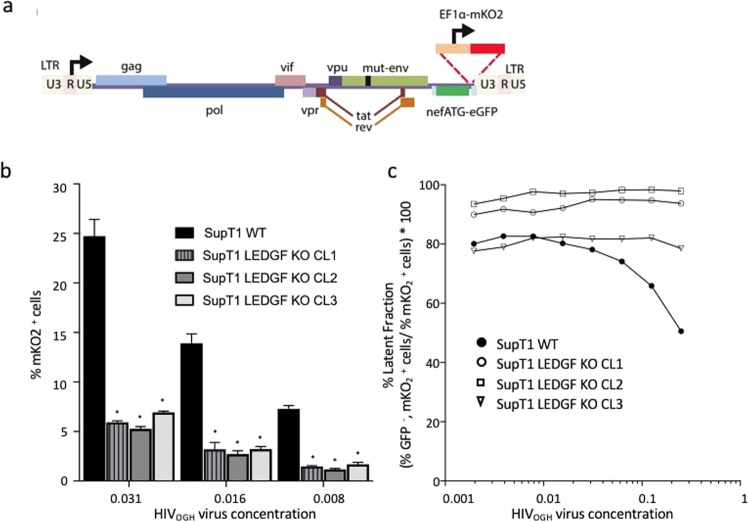
LEDGF depletion results in an increase of the silent HIV reservoir. (**a**) Schematic representation of the dual-colored VSV-G pseudotyped HIV_OGH_ reporter virus carrying an eGFP cDNA driven by the viral LTR promoter in the *nef* position and an entire constitutive transcriptional unit (EF1a-mKO2) inserted downstream. (**b**) HIV provirus integration is greatly diminished in the absence of LEDGF/p75 when compared to SupT1_WT cells, as evidenced by the lower %mKO2 positive cells for a given virus concentration. Error bars represent SD. Student’s *t* test was performed using GraphPad Prism 7.0 software. Sample means were considered significantly different from the WT control at p < 0.05 (*). (**c**) The latent fraction for the respective cell lines for different virus concentrations. LEDGF KO increases the fraction of silently infected cells ((% eGFP^−^, mKO2^+^ cells)/% mKO2^+^ cells) ∗ 100. eGFP, Enhanced Green Fluorescent Protein; mKO2, Mutant Kusubira Orange 2. The experiment was performed three times. The plots are representatives of one of three independent infection experiments.

HIV-1 and other lentiviruses preferentially integrate into actively-transcribed regions of the genomic DNA in the presence of LEDGF/p75^42^. It was recently reported that depletion of LEDGF/p75, known to reduce integration and to alter the integration profile of residual HIV integrants^26,43,44^ also demonstrates a more quiescent phenotype that is refractory to re-activation^43,44^. To examine the degree of lentiviral integration and to assess the ratio of productive versus latent transduction, we used the single-round, orange-green HIV-1 (Fig. 6a; HIV_OGH_) double reporter virus that allowed quantification of actively-infected (eGFP^+^, mKO_2_^+^) and latently-infected (eGFP^−^, mKO_2_^+^) cell populations^45,46^. SupT1_WT and the respective SupT1_LEDGF KO cell lines were challenged with a dilution series of HIV_OGH_. The percentage of infected cells was evaluated on day 3 post infection, discriminating productively infected cells (eGFP^+^, mKO_2_^+^) from the latently-infected populations (eGFP^−^, mKO_2_^+^). Depletion of LEDGF/p75 reduced the overall transduction efficiency by 4-fold (compare % mKO_2_^+^ cells for different vector dilutions; Fig. 6b)^44,47^. Moreover, in line with earlier data^43^, the larger portion of proviruses were latent (latent fraction = percent eGFP^−^/mKO_2_^+^ cells over total mKO_2_^+^ cells; Fig. 6c). In SupT1_WT cells, higher viral concentrations result in more productively infected cell numbers (lower % of latently infected cells; Fig. 6c). For the respective LEDGF/p75 CRISPR-KO cell lines between 80–95% of the cells were quiescently infected. Although the relative percentages are different between the KO clones, the pattern persisted over a series of viral dilutions (Fig. 6c).

It is known that CRISPR/Cas-based knock-out can result in off-target genomic disruptions. To establish that the phenotype observed in the LEDGF KO lines was not a consequence of clonal bias or off-target event, we reintroduced wild-type LEDGF/p75 in SupT1_LEDGF KO clone 3. Rescue of LEDGF/p75 protein expression was corroborated by flow cytometry and Western blot (SupT1_LEDGF KO clone 3 BC; Supplemental Fig. 5a,b, respectively). The cell line was infected with the laboratory strain HIV_NL4.3_ and showed an increase in viral replication comparable to wild-type (Supplemental Fig. 5c), albeit delayed. In parallel, we challenged the LEDGF/p75-complemented cells with the double reporter HIV_OGH_ virus and showed that LEDGF/p75 reintroduction resulted in comparable % mKO_2_^+^ cells as SupT1_WT (Supplemental Fig. 5d). In line, the latent fraction showed a comparable pattern as observed in wild-type cells (Supplemental Fig. 5e).

### HIV-1 does not replicate in SupT1_LEDGF D366N cells and residual HIV-1 integrants adopt a latent phenotype

**Figure 7:**
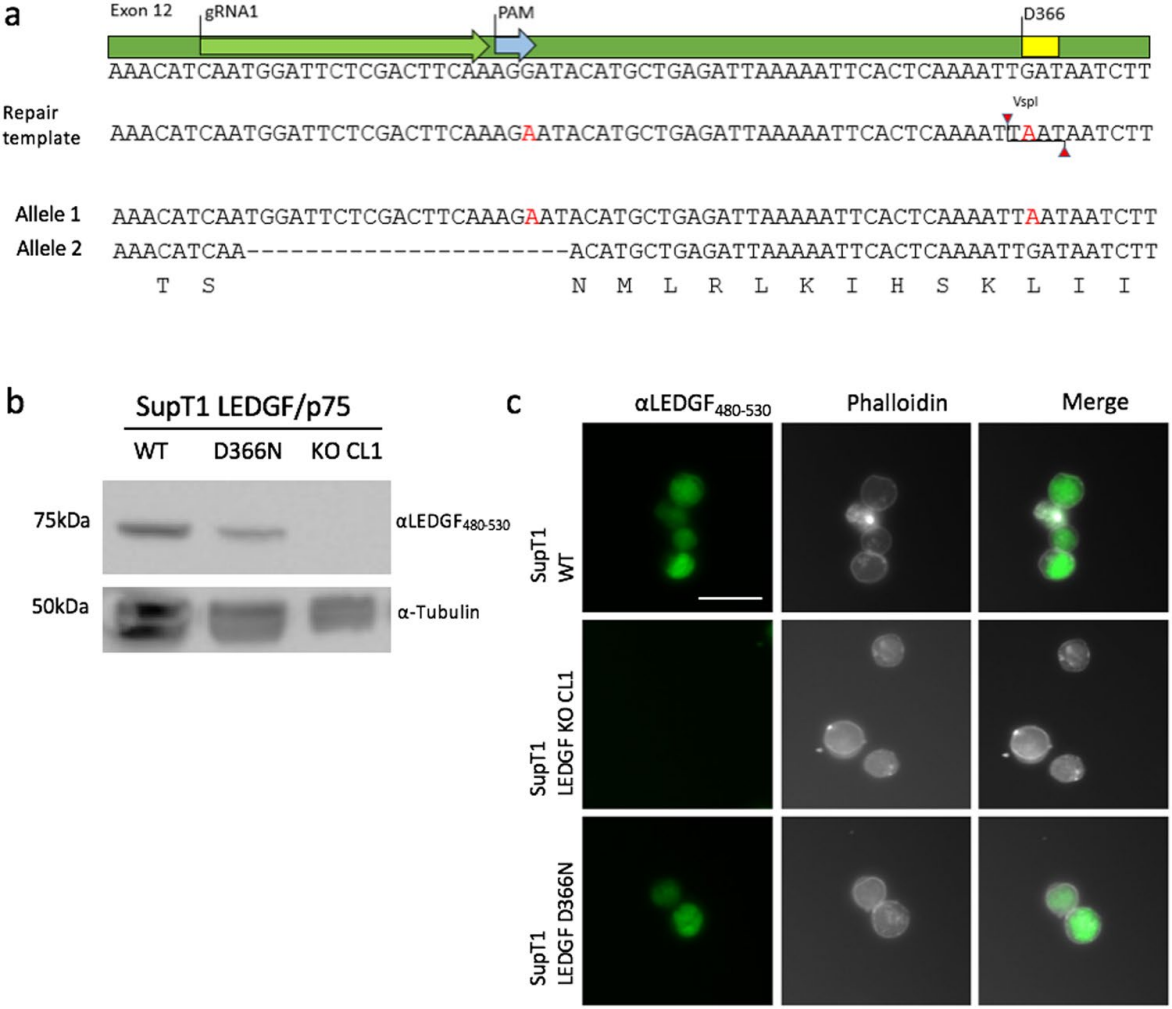
Characterization of SupT1_LEDGF D366N. (**a**) Sanger sequencing results showing consensus between HDR template and allele 1. The HDR template is shown with red characters indicating the sites of nucleotide substitutions, generating a *Vsp*I restriction site at D366N and deleting the PAM site. The sequence for allele 2 shows a 22-nucleotide deletion gRNA1 (“−” indicates deletion), resulting in KO of the *PSIP1* allele. (**b**) Western blot analysis for the SupT1_LEDGF D366N clone using C-terminal specific LEDGF antibody, αLEDGF_480–530_. SupT1_WT and KO cells are included as controls. (**c**) Immunocytochemical analysis of LEDGF D366N protein (green) in SupT1_LEDGF D366N cells (bottom panel) recapitulates that of LEDGF/p75 in SupT1_WT cells (top panel). Phalloidin (white) was used to stain F-actin. Scale Bar: 10 µm.

To determine whether D366N in SupT1 cells also confers resistance to HIV-1, Cas9-expressing SupT1 cells were electroporated with gRNA1 plasmid along with the repair template and monoclonally expanded. Out of library of 200 clones, a single clone carrying the D366N mutation was identified through biallelic sequencing. Similarly to the HEK293T mutant, the clone contained one mutant allele with the sequence identical to the repair template and a second allele with a 22-nucleotide deletion (Fig. 7a). Western blot analysis showed LEDGF D366N at the same molecular height as LEDGF WT (Fig. 7b). The localization of LEDGF D366N protein was confirmed to be nuclear as for SupT1_WT condition (Fig. 7c).

**Figure 8:**
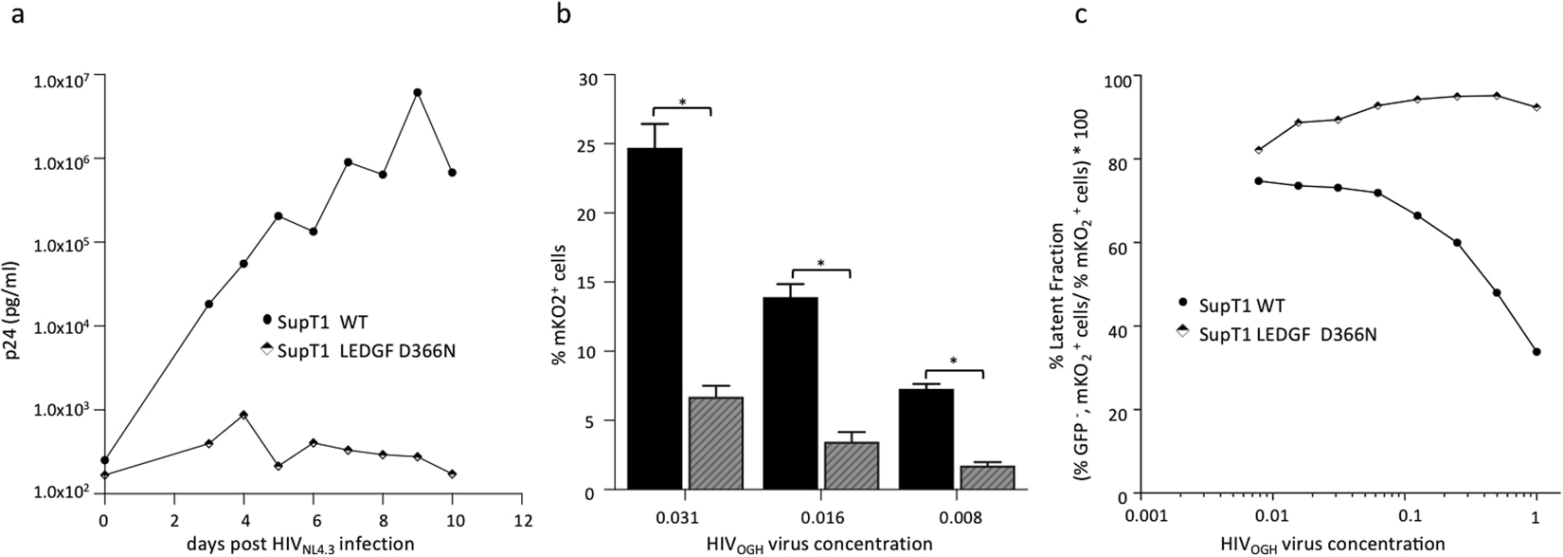
HIV replication is hampered in SupT1_LEDGF D366N cell line as compared to the SupT1 WT cell line. (**a**) HIV-1 replication assay. The cell lines were challenged with the laboratory HIV_NL4.3_ strain at a final concentration of 5.0*10^2^ pg p24. Replication was monitored by daily sampling of the p24 levels in the cell culture supernatant. (**b**) Introduction of the D366N mutation in LEDGF/p75 renders SupT1 cells refractory to transduction as shown by a 4-fold decrease in %mKO2 expressing cells for a given VSV-G pseudotyped HIV_OGH_ virus concentration. Student’s *t* test was performed using GraphPad Prism 7.0 software. Sample means were considered significantly different from the WT control at p < 0.05 (*). (**c**) In addition to the lower transduction efficiency observed for SupT1_LEDGF D366N, the latent fraction is greater when compared to SupT1_WT cells, even at higher concentrations of HIV_OGH_ virus. eGFP, Enhanced Green Fluorescent Protein; mKO2, mutant Kusubira Orange 2. The plots represent one of three independent infection experiments.

Next, we challenged the SupT1_LEDGF D366N cells with HIV_NL4.3_ virus. The cells carrying the D366N mutation demonstrated resistance to the HIV challenge, showing no viral replication following infection with the HIV_NL4.3_ laboratory strain (Fig. 8a). Also, we challenged the SupT1_LEDGF D366N mutant cells with the HIVOGH double-reporter virus to determine whether the D366N mutation affects the integration and integrant distribution to a latent proviral pool. In line with what we observed in the SupT1_LEDGF KO cells, transduction efficiency was about 4-fold lower in the SupT1_LEDGF D366N cells compared to the SupT1_WT cells (compare % mKO_2_^+^ cells for different vector dilutions; Fig. 8b). Besides a lower transduction efficiency, a higher proportion of the remaining infections in SupT1_LEDGF D366N cells was transcriptionally silent, even at higher concentrations of HIV_OGH_ virus (eGFP^−^, mKO2^+^ cells / total mKO2^+^ cells; Fig. 8c). For the SupT1_LEDGF D366N cells about 95% of the cells were quiescently infected, which was corroborated for a wide range of viral dilutions, whereas the latent fraction in SupT1_WT cells dropped substantially at higher virus concentrations (Fig. 8c).

### LEDGF D366N mutation confers resistance to HIV-1 challenge even at high MOI

**Figure 9:**
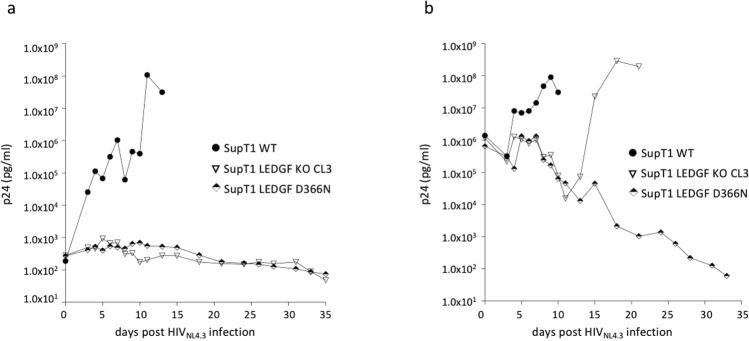
HIV replication remains hampered in SupT1 LEDGF D366N cell line at high MOI as compared to the SupT1 WT and SupT1 LEDGF KO cell line. (**a**) HIV-1 replication assay at low MOI. The cell lines were challenged with the laboratory HIV_NL4.3_ strain at a concentration of 1.5*10^6^ pg p24 for 2 h. The cells were then washed to remove the virus to result in 5.0*10^2^ pg/ml p24 final concentration and monitored for replication. (**b**) HIV-1 replication assay at high MOI. The cell lines were challenged with the laboratory HIV_NL4.3_ strain at a final concentration of 1.5*10^6^ pg p24. Replication was monitored by daily sampling of the p24 levels in the cell culture supernatant. Each plot represents one of three independent infection experiments.

In an effort to test whether the HIV-1 virus could escape and grow resistant by adapting to the LEDGF D366N point-mutation, we performed longer-term HIV_NL4.3_ replication experiments at low and at high MOI and monitored replication for more than 4 weeks (Fig. 9). For low MOI experiments, we pelleted 5*10^5^ of SupT1 cells (SupT1_WT, SupT1_LEDGF KO clone 3 and SupT1_LEDGF D366N, respectively) and challenged these with 1.5*10^6^ pg p24 of HIV_NL4.3_ for 2 hrs before washing the cells, resulting in a final dose of approximately 2*10^2^ pg p24/ml. While p24 concentration readily increased in SupT1_WT cells, HIV replication was not detected in both SupT1_LEDGF KO clone 3 and SupT1_LEDGF D366N within 4 weeks (Fig. 9a). For the high MOI experiments, 5*10^5^ SupT1_WT, SupT1_LEDGF KO clone 3 and SupT1_LEDGF D366N cells were challenged with 5*10^6^ pg p24 HIV_NL4.3_ but were not washed to remove the initial inoculum, resulting in a final dose of 1*10^6^ pg p24/ml. Although the p24 concentration in the supernatant was high compared to p24 concentration at day 0 (Fig. 9a), HIV replication was detected from day 5 onwards in SupT1_WT cells, whereas no replication was detected in SupT1_LEDGF KO clone 3 and SupT1_LEDGF D366N, where p24 values remained constant or declined. On day 15, HIV replication was also detected in SupT1_LEDGF KO clone 3 cells (Fig. 9b), which is in line with an earlier report, where HRP-2 was shown to take over the tethering function of LEDGF/p75 in LEDGF depleted cells^48^. However, in SupT1_LEDGF D366N no viral replication was detected when monitored for 33 days post infection, indicating that the more nuanced approach using gene editing renders cells more resistant to HIV escape than the ablation of LEDGF/p75.

## Discussion

The definitive success of the antiretroviral drugs at controlling HIV replication has turned the once deadly HIV infection into a manageable, chronic illness. However, even though it revolutionized the treatment of HIV disease, cART cannot eradicate HIV virus and thus does not provide a cure. Following treatment interruption, viral replication rebounds despite years of treatment. Reactivation of the latent HIV reservoir necessitates a lifelong, daily medication regimen with considerable unwanted complications, such as emergence of viral resistance, chronic inflammation and immune dysfunction, accelerated aging, cardiovascular disease, liver and renal failure, neurocognitive dysfunction, and HIV related malignancies^49–52^. Given the limitations and complications of antiretroviral therapy, curative strategies to treat HIV disease are considered high-priority. Two main approaches have garnered considerable attention over the past decade: purging of the latently infected reservoir using latency reversing agents and protection of uninfected HIV target cells through site-specific gene targeting or gene therapy^53–55^. A tropism-independent approach to rendering CD_4_^+^ T cells refractory to HIV infection is to target the integration step of the HIV replication cycle by uncoupling the viral IN from the host integration facilitator LEDGF/p75.

LEDGF/p75 knock-down experiments have previously shown that HIV replication is greatly impaired, however, the residual functional activity, even at very low expression levels, eventually results in HIV breakthrough in spreading infection studies^56–58^. More recently, a number of stable LEDGF knock-out cell lines were generated by different site-specific gene targeting approaches demonstrating profound reduction in spreading HIV-1 replication, exceeding the replication delays observed for stable knock-down CD_4_-T cell lines^14,26^ establishing the role of LEDGF/p75 as a co-factor for efficient viral propagation. The knock-out approach has shown no deleterious phenotype in mature T-cells, however, a recent study demonstrated that knocking out *PSIP1 in vivo* leads to hematopoietic defect, showing a two-fold reduction in peripheral lymphocytes as well as a lower numbers of HSCs, splenocytes, and thymocytes^59^. Moreover, LEDGF/p75 has been shown to interact with numerous cellular proteins via its IN binding domain (IBD), ruling out complete *PSIP1* ablation as a potential therapeutic strategy. Interestingly, structural characterization of the IBD interactions showed that the cellular binding partners of LEDGF/p75 interact with the IBD through an intrinsically unstructured IBD-binding motif (IBM), whereas IN is structurally prearranged and binds the IBD via a larger hydrophobic surface area^24,35^. Mutation of the protruding aspartic acid residue D366 in the LEDGF IBD specifically disrupts binding of the HIV IN dimer^36,37^, leaving the binding of cellular binding partners unaffected. We used CRISPR/Cas9 gene editing to generate stable *PSIP1* (LEDGF) knock-out cell lines as well as a *PSIP1* D366N mutant line designed to disrupt the successful binding of HIV IN.

Single-amino acid substitution was achieved by generating a double-stranded break using CRISPR/Cas9 and relying on homology directed repair pathway to recombine with the provided repair template oligo carrying the D366N mutation. The editing process proved inefficient with HDR taking place in approximately 12% of cells under HDR-promoting experimental conditions. This low efficiency is most likely due to the distance between the site of the DSB and the D366 residue being 39 bp, highlighting the clear limitation of the CRISPR/Cas system in its dependence on the PAM site. It has previously been reported that the frequency of HDR is negatively correlated with the distance from DSB^38^. The mutation of the PAM site, 5 bp way from DSB, which was also encoded in the repair oligo, had a 10-fold increase in frequency compared to D366N (data not shown). A modified Cas9 with an alternative PAM (NGAN) site has been synthesized able to generate a cut 3 bp away from our desired location^60^. This new Cas9 mutant could potentially increase the editing frequency, provided that the gRNA targeting efficiency is retained, making D366N knock-in strategy more applicable clinically. Alternatively, Cas9 RNPs have been shown to generate knock-in edits in primary T cells^61^. Even though currently the efficiency of the knock-in editing process is significantly lower than that of knock-outs, as technology evolves, we expect this approach will become more feasible.

We were able to isolate a clone of each modified cell line carrying the desired mutation in one *PSIP1* allele, while the other allele was knocked-out. The HEK293T_D366N mutant clone demonstrated successful uncoupling from HIV-IN in the co-localization study, whereas LEDGF/p75 interaction partners JPO2 and IWS1 retained their binding ability. The SupT1_D366N mutant clone did not support HIV replication when challenged with HIV_NL4.3_ (Fig. 8a), in line with previously reported data^25^. These data were corroborated using the double reporter HIV_OGH_: infection of SupT1_LEDGF KO and D336N cells was 4–5-fold less efficient compared to SupT1_WT cells (% mKO_2_^+^ cells in Figs 6 and 8, respectively). In addition, the remaining HIV_OGH_ viruses that were able to integrate in the absence of LEDGF/p75 were shown to be predominantly quiescent, limiting the probability of mounting a productive infection, in line with earlier data^43^.

The ability of the CRISPR/Cas system to generate a true genetic knock-out allows for functional interrogation in a well-defined genetic system. In both the HEK293T and SupT1 cells, the disruption in the IBD region within exon12 using CRISPR/Cas resulted in efficient knock-out. Transient transfection with gRNA-loaded CRISPR/Cas plasmid resulted in 94% LEDGF/p75 protein knock-out in HEK293Ts and 88% in SupT1 cells after electroporation and sorting for successfully transfected cells as determined by the FACS analysis of polyclonal populations. We could not demonstrate residual or truncated LEDGF/p75 protein in the gRNA1 CRISPRed LEDGF KO clones using Western analysis or immunocytochemistry. Analysis of *PSIP1* expression in the isolated clones revealed that mRNA levels were also significantly reduced (Figs 2d and 5c). Frame-shift mutations in coding genetic regions have been shown to trigger nonsense-mediated mRNA decay (NMD)^62^, which serves as mRNA quality check-point mechanism to prevent translation of transcripts with a premature stop codon thus eliminating the potential toxic effects of truncated proteins. The sequencing results for two of the three knock-out clones in both HEK293T (clone 1 and 3) and SupT1 (clone 1 and 2) cells showed frame-shift mutations resulting in a premature stop codon in both alleles, corroborating the theory of NMD as the probable degradation pathway. SupT1_KO clone 3 showed a premature stop codon in one allele and an in-frame 8 amino-acids deletion in the other, yet a 98% depletion of mRNA was observed, suggesting an alternative mRNA decay pathway. Similarly, the HEK293T_KO clone 2, which was determined through targeted resequencing to be triploid in the CRISPR/Cas-edited region (Supplemental Fig. 1), showed a premature stop codon in two of the alleles and a single amino acid deletion (Q353) in the third allele. The mRNA was 70% depleted relative to the WT *PSIP1* mRNA levels. The 30% expression level may indicate that the mRNA of the delQ353 allele was stable, however, no LEDGF protein was detected. Q353 is part of a highly ordered helix bundle consisting of 4 helices in the IBD of LEDGF/p75 and modeling analysis predicts the sole deletion of Q353 to result in a shift in the helical pattern, destabilizing the entire structure, resulting in failure to fold properly, and targeting the mutant for degradation.

Analyses of spreading HIV-1 infection in the SupT1 stable LEDGF KO cell lines showed an HIV replication defect, corroborating the previously reported replication delays in TALEN-edited Jurkat cells and in other human CD_4_^+^ T cell lines with a stable shRNA-mediated knockdown^14,26,44^. Moreover, when using a double-reporter single round HIV_OGH_ virus, we demonstrated 4–5 fold reduced transduction efficiency (% mKO_2_ positive cells) in LEDGF D366N cells. Additionally, for the remaining integrated proviruses, the fraction of mKO_2_^+^ cells that were GFP negative (latent fraction) was higher in the SupT1_LEDGF D366N cells (Fig. 8c), in line with what we observed for SupT1_LEDGF KO clones (Fig. 5c) and previously reported data for LEDGF/p75 depleted and LEDGIN treated cells^43^. One could argue that the more nuanced approach of gene editing carries the inherent danger of easier HIV-1 escape by adaptation of HIV IN to the point-mutated LEDGF D366N. However, long-term replication assays using the laboratory HIV_NL4.3_ strain at high MOI in SupT1_WT, SupT1_LEDGF KO and SupT1_LEDGF D366N cells showed HIV replication in SupT1_WT and SupT1_LEDGF KO cells, albeit with a delay in the SupT1_LEDGF KO (Fig. 9b). The latter is in line with other reports and can be attributed to HRP-2^44^. Like LEDGF/p75, HRP-2 carries a PWWP and an integrase binding domain (IBD), however HRP-2 binds IN with lower affinity and can only substitute for LEDGF/p75 when LEDGF/p75 is ablated. Interestingly, no viral replication was detected in SupT1_LEDGF D366N cells that were monitored for up to 33 days post infection, demonstrating that the gene editing approach rendered cells more resistant to HIV escape than the ablation of LEDGF/p75. A possible explanation for the latter results is that in SupT1-LEDGF D366N cells the D366N mutation prevents HIV IN to bind its natural tether, leaving HRP-2 as the privileged binding partner. But with the LEDGF D366N protein still occupying its cognate chromatin sites the HRP-2 protein is prevented from docking. In the absence of LEDGF/p75 (SupT1_LEDGF KO), however, LEDGF/p75 chromatin binding positions are accessible for HRP-2 leading to replication.

With ZFN-based CCR5 knock-out as first-line gene therapy target in phase II clinical trials^63^, the gene therapy approach is gaining traction as a promising curative therapeutic treatment for HIV^64^. The CRISPR/Cas system has proven time and again to be the most efficient, adaptable and scalable tool for genetic ablation and editing^65,66^. The observation that editing LEDGF/p75 did not result in evident defect in laboratory T cells^14,26^ opens perspectives to combine *PSIP1* and *CCR5* for combinatorial site-specific gene targeting. Indeed, an arrayed editing of HIV entry co-receptors in combination with LEDGF/p75 was validated as a protective strategy against HIV-1 in primary T-cells^14^. Although disruption of CCR5, CXCR4 and LEDGF/p75 appears to be tolerated by CD_4_^+^ T-cells *in vitro*, ablation of any gene would need to be thoroughly studied before being considered for any cell-based therapy. In our study, the precise editing of LEDGF/p75 using the D366N knock-in approach circumvents any potential deficiency stemming from LEDGF/p75 disruption, while protecting cells against HIV infection. Similar efforts can be employed in generating CCR5 and CXCR4 knock-ins refractory to HIV.

## Methods

### Cell culture

Cells were cultured in a humidified atmosphere containing 5% CO_2_ at 37 °C. SupT1 (T-cell lymphoblastic lymphoma; provided by the National Institutes of Health (NIH) Reagent Program, NIH, Bethesda, MD) and were cultured in RPMI (GIBCO) with 10% v/v FCS (Sigma-Aldrich). HEK293T cells (gift from O. Danos, Evry, France) were cultured in Dulbecco Modified Eagle Medium (DMEM, GIBCO) with 5% v/v FCS (Sigma-Aldrich) and 0.01% v/v gentamicin (GIBCO).

### CRISPR/Cas tools and guide validation

Guide RNAs were generated using the CRISPR design and analysis tool from the Massachusetts Institute of Technology (http://crispr.mit.edu) and ligated into CRISPR/Cas9 plasmids as previously described^67^. Briefly, the 24 bp forward and reverse primers including the 20-bp target sequence and BbsI sticky ends were annealed and ligated into pSpCas9(BB)-2A-GFP (pX458) plasmid (Addgene plasmid #48138), a gift from Feng Zhang^67^. Primer sequences are shown in Supplemental Table S1. For transfection of cell lines that constitutively express Cas9 protein, a smaller, guideRNA-scaffold only plasmid encoding the eGFP reporter to facilitate cell sorting was generated. To that end, the Cas9 ORF was removed from pX330 plasmid (Addgene #42230, gift from Feng Zhang) using HindIII and EcoRI. The eGFP under SFFV-promoter sequence was then ligated into the modified pX330 backbone resulting in a plasmid 4267 bp in size named pX321-eGFP. For production of Cas9 lentiviral vector transfer plasmid, the gRNA scaffold and the U6 promoter were removed from the lentiCRISPR v2 plasmid (Addgene #52961, gift from Feng Zhang) by digesting it with Pst1 and EcoRI and a 207 bp adaptor sequence was inserted resulting in a plasmid 12674 bp in size named LentiCRISPRv2+ adaptor. The resulting lentiviral vector expresses Cas9 and a puromycin resistance cDNA, and is referred to as LV_Cas9-I-PuroR. All cloning steps were sequence verified.

For guide RNA validation experiment, guides 1, A and B were cloned into pX330 plasmid individually and transfected into HEK293T cells in equimolar amounts in pairs (gRNA 1+ A; gRNA 1+ B) using polyethylene-imine (PEI) transfection agent. The cells were allowed to expand for 5 days before collection for Western blot and genomic DNA analysis. To assess the presence of the segmental deletion in the genome, a pair of primers was designed to anneal outside the regions recognized by gRNA1 and B (Supplemental Table S1 and Fig. 1b). The truncation in the genome was detected by assessing the PCR product size observed on an agarose gel. The amplicon size of 581 bp would indicate either a WT sequence or a sequence that has been cut with a single gRNA. For a segmental deletion, a band size of 506 bp for guide pair 1+ A or 283 bp for guide pair 1+ B would be observed on an agarose gel, indicating that both guides within a pair are functional (Fig. 1c).

### Generation of stable knock-out and D366N knock-in cells lines

HEK293T_LEDGF KO cells were generated by transient transfection of pX458 plasmid containing gRNA1 using branched polyethylene-imine (PEI) transfection agent (Sigma-Aldrich). Cells were sorted for GFP using Fluorescence Activated Cell Sorting (Bio-Rad S3) 48 h after electroporation and were allowed to expand for one week before seeding for monoclonal expansion into a 96 well plate. The individual clones were screened by Western blot and Immunocytochemical analysis.

To generate LEDGF D366N cells, ssODN repair oligo from IDT (Leuven, Belgium) was added at 10pM concentration to the transfection mix. SCR7 compound (1 µM) (ApexBio A8705-5) was added to the cells immediately after transfection and left on for 24 h. Cells were sorted for GFP using Fluorescence Activated Cell Sorting (Bio-Rad S3) 48 h after electroporation and were allowed to expand for one week before seeding for monoclonal expansion into a 96 well plate. The individual clones were screened by isolating genomic DNA and amplifying the D366 region of 581 bp using primer set reported in Supplemental Table S1. The presence of N366 was detected by VspI digest.

To generate SupT1_LEDGF KO and D366N cells, SupT1-Cas9 cells were generated first to constitutively express Cas9 nuclease using LV_Cas9-I-PuroR and subsequent puromycin selection. For electroporation of pX321-GFP into SupT1-Cas9 cells, 1 × 10^6^ cells were resuspended in 100 μl serum-free RPMI medium with 10 μg of plasmid. Cells were transferred into an electroporation cuvette and electroporated (175 V, 6 pulses) using Gene Pulser Xcell system (Bio-Rad). Cells were sorted for GFP using Fluorescence Activated Cell Sorting (Bio-Rad S3) 48 h after electroporation and were allowed to expand for one week before seeding for monoclonal expansion into a 96 well plate. For HDR experiments, ssODN repair oligo from IDT (Leuven, Belgium) was used at 10pM concentration along with pX321-GFP plasmid. SCR7 compound (1 µM) (ApexBio A8705-5) was added to the cells immediately after transfection and left on for 24 h. The individual clones were screened by isolating genomic DNA and amplifying the D366 region of 581 bp using primer set reported in Supplemental Table S1. The presence of N366 was detected by VspI digest.

#### Western blot analysis

Western blotting was performed as described previously^58^. Briefly, cells were seeded at 3*10^5^ cell/well in 6-well plates and grown overnight. Cells were washed twice with 1x PBS and subsequently crude lysates were prepared using 1% sodium dodecyl sulfate (SDS, Sigma-Aldrich). Lysates were boiled for 2 minutes, and gDNA was sheared through an insulin-needle. Protein concentrations were determined using a bicinchoninic acid (BCA, Pierce BCA Protein Assay kit, ThermoScientific). In total, 20 μg protein was used for electrophoresis on a 4–15% Tris-glycine gel (Criterion TGX Precast Midi Protein Gel). Next, proteins were electroblotted on a polyvinylene difluoride membrane (PVDF; BioRad). After protein transfer, the membrane was blocked using 5% milk-powder in 0.1% PBS-Triton. LEDGF/p75 was detected using rabbit anti-human LEDGF polyclonal antibody at 1:500 ON at 4 °C (A300–848A and A300–847A; Bethyl Laboratories Inc., Montgomery, TX). After 4 wash steps in 0.1% PBS-Triton the membrane was incubated with the secondary antibody at 1:10000 for 1 h (goat anti-rabbit conjugated with horse-radish peroxidase (HRP), Dako). Flag-tagged proteins were detected using monoclonal anti-flag M2 antibody produced in mouse (1:1000; F1804, Sigma-Aldrich) and a secondary goat anti-mouse HRP (1:10000, Dako). Equal loading was verified using either α-tubulin at 1:5000 (T-9026; Sigma-Aldrich, St Louis, MO) or β-tubulin at 1:5000 (T-8328; Sigma-Aldrich, St Louis, MO). Protein signals were detected using Western ECL substrate (BioRad) on a LAS-3000 Imaging System (Fuji).

### Immunocytochemistry and co-Immunoprecipitation Analysis

For immunocytochemistry analysis, 3*10^4^ HEK293T cells were seeded in Lab-Tek^TM^ microscopy chamber slides (ThermoScientific). After 24 h, the cells were fixed with 4% paraformaldehyde and stained with LEDGF antibody at 1:400 (A300–848A, Bethyl Laboratories Inc, Montgomery, TX). Alexa Fluor® Phalloidin 555 (A34055, ThermoFisher) was used as F-actin counterstain.

For protein co-localization and coIP experiments plasmid constructs that expressed 3xFlag-tagged versions of IWS1, JPO2 and HIV IN, pCHMWS INs-3xFlag-I-PuroR, pCHMWS 3xFlag HsIWS1-I-HygroR, pCHMWS 3xFlag JPO2-I-HygroR, were described by Tesina *et al*.^24^. Plasmid constructs to express LEDGF and LEDGF D366N, pGAE-SFFV LEDGF BC-I-BsdR and pGAE-SFFV LEDGF BC D366N-I-BsdR, respectively were previously described^68^.

For co-localization experiments, WT and D366N HEK 293 T cells were transfected with plasmid constructs carrying Flag-tagged JPO2, IWS1, or HIV-IN 30 h prior to fixation. Flag tag was detected using Flag antibody at 1:1000 for 24 h (F4725, Sigma). Samples were analyzed using LSM 510 META imaging unit (Carl Zeiss, Zaventem, Belgium) as described before^39^.

CoIP was essentially performed as described earlier with small modifications^69^. Briefly, 5.8 × 10^6^ HEK293T_LEDGF KO clone1 cells were plated in a 8.5 cm dish and transfected the next day with plasmid that expressed either LEDGF or LEDGF D366N (20 μg for pGAE-SFFV LEDGF BC-I-BsdR and pGAE-SFFV LEDGF BC D366N-I-BsdR, respectively) together with a plasmid construct that expressed the LEDGF cellular binding proteins, IN-3xFlag, 3xFlag-IWS1 or 3xFlag-JPO2 (20 μg pCHMWS INs-3xFlag-I-PuroR and 30 μg pCHMWS 3xFlag HsIWS1-I-HygroR, pCHMWS 3xFlag JPO2-I-HygroR, respectively) using branched PEI. The next day, cells were washed with phosphate buffered saline (1x PBS) and lysed in 700 μl cytoplasmic lysis buffer/plate (20 mM Tris-HCl pH 7.4, 150 mM NaCl, 0.1%[v/v] IGEPAL, 1 mM DTT and 1X Complete Protease Inhibitor Cocktail [Roche, Germany]) for 10 min on ice. Nuclei were pelleted at 4000xg and lysed in 500 μl nuclear lysis buffer (cytoplasmic lysis buffer with 400 mM NaCl) for 60 min on ice. The lysate was cleared by centrifugation at 18000 × g and incubated with 25 μl Anti-FLAG®M2-agarose affinity resin (Sigma-Aldrich). The beads were pelleted by centrifugation (30 s, 1000 × g, 4 °C) and washed 3 times in 600 μl 400 mM NaCl cytoplasmic lysis buffer. Immuno-precipitated protein was eluted with 40 μl SDS-PAGE loading dye and visualized by Western blotting.

### Sequencing of CRISPR-targeted genomic region

The genomic DNA was isolated using GenElute™ Mammalian Genomic DNA Miniprep Kit (G1N70, Sigma). The PCR fragments (581 bp) were amplified using Fwd and Rv primers (Supplemental Table S1) with the following conditions: denaturing for 30 sec at 95 °C, followed by 32 cycles of 10 sec at 95 °C, 30 sec at 58 °C, and 30 sec at 72 °C, followed by 5 min of final extension at 72 °C. The amplicons were then cloned into the pCRII-TOPO plasmid (Invitrogen, Merelbeke, Belgium) and sequenced with primers M13-Fwd and M13-Rev (Supplemental Table S1).

### MiSeq targeted resequencing analysis of CRISPRed genomic region

The region of interest was amplified with Q5 polymerase (M0491S, NEB) using MiSeq primers appended with overhang adapter sequence (Supplemental Table S1), resulting in a 260 bp amplicon. The amplicons were then indexed with multiplexing indices in the subsequent limited-cycle PCR step (Nextera® XT Index Kit, FC-131-1001, Illumina). The products were purified (28106, Qiagen), quantified and diluted before loading into MiSeq cartridge. MiSeq Reagent Kit (300-cycles – PE) MS-102-1001 was used for sequencing in the MiSeq System (SY410-1003, Illumina). The sequencing reads where processed using fastq-MCF (ea-utils) and a quality control was performed with fastQC. Subsequently the reads were mapped with Bowtie2 to the Human genome (hg38). Integrative Genomics Viewer (IGV) was used for further analysis, together with a number of custom-made scripts.

### Quantitative PCR

Quantification of *PSIP1* mRNA levels was performed as described previously^47^. β-Actin was used as endogenous house-keeping control (TaqMan Control Reagent; Applied Biosystems). All samples were run in triplicate for 3 minutes at 95 °C followed by 50 cycles of 10 seconds at 95 °C and 30 seconds at 55 °C. Data were analyzed with iQ5 Optical System Software (BioRad, Nazareth, Belgium).

### HIV Viral replication assay

HIV replication experiments were performed at low and at high MOI. For standard experiments (low MOI), we pelleted 5*10^5^ of SupT1 cells (SupT1_WT, SupT1_LEDGF KO clone 3, SupT1_LEDGF KO clone3 BC or SupT1_LEDGF D366N) and challenged these with 1.5*10^6^ pg p24 of HIV_NL4.3_. After 2 hrs of infection, cells were washed twice with 1xPBS and resuspended in 5 ml of culture medium in T25 flasks resulting in a final dose of around 5*10^2^ pg p24/ml. In addition, high MOI experiments were performed using 5*10^5^ cells (SupT1_WT, SupT1_LEDGF KO clone 3 and SupT1_LEDGF D366N) seeded in 5 ml of medium in T25 flasks and infected with 5*10^6^ pg p24, resulting in a final dose of 1*10^6^ pg p24/ml. The viral inoculum was not washed. HIV replication was monitored by daily sampling of the supernatants and subsequent quantification by p24 ELISA (Alliance HIV-1 p24 ELISA kit: Perkin Elmer). Cells were split 1/2 every 2 days.

### Multi-Colored reporter virus (HIV_OGH_), Lentiviral Vector Production and Transduction

A variant of the recently described LAI-based double reporter virus was used, where a constitutive and an LTR-driven reporter are simultaneously measured to study the latent reservoir^45,46,70^. This orange-green HIV-1 (OGH) reporter virus variant encodes LTR-driven enhanced Green Fluorescent Protein (eGFP) together with a constitutively active EF1alpha promoter driving monomeric Kusabira-Orange2 (mKO2) expression. Through the constitutively active EF1α promoter, all cells carrying an integrated provirus express the mKO2 reporter; eGFP expression is driven by the active LTR promoter is a marker for an active provirus and are productively infected. The mKO positive cells that do not express eGFP are considered to contain silent or latent provirus. HIV_OGH_ was prepared as described earlier with small modifications^43^. Briefly, vesicular stomatitis virus G (VSV-G)-pseudotyped single-round viruses were generated by double transfection of HEK293T cells with a plasmid encoding a single round HIV clone pOGH together with a VSV-G protein encoding plasmid (pVSVG), using branched PEI (MW 25,000; Sigma-Aldrich). Medium was replaced 6 h post transfection and supernatant collected after 72 h by filtration through a 0.45 μm pore membrane (MillexHA, Millipore). The virus was concentrated using an Amicon 50 kDa cut-off column (Millipore) by centrifugation at 3000 × g, DNase (Roche) treated (at 10 U/μg plasmid DNA) for 2 hrs and stored at −80 °C. For transduction, cells were seeded at 25,000 cell/well and infected for 3 days in 48-well plates (10% FCS, 0.01% gentamicin RPMI) yielding an infection rate < 40% positive cells, as monitored by FACS analysis using a MACS Quant VYB FACS analyzer (Miltenyi Biotech GmbH), ensuring single-copy integration. Cells were washed twice with Phosphate Buffered Saline (PBS) 72 h post infection to remove residual virus and reseeded. FACS samples were taken every 2 days to monitor reporter gene expression.

For lentiviral vector production, triple plasmid transfection was done with the transfer plasmid (LentiCRISPR v2 + adaptor) together with the p8.91 packaging plasmid and pVSVG in ratio of 15:10:5, respectively, using branched PEI (MW 25,000; Sigma-Aldrich). Medium was replaced 24 h post transfection and supernatant collected after 48 h by filtration through a 0.45 μm pore membrane (MillexHA, Millipore). The lentiviral vector was concentrated using an Amicon 50 kDa cut-off column (Millipore) to 1 ml and stored at −80 °C.

## Supplementary information

Supplemental Figures
