# Supplementary material for: Targeted editing of the PSIP1 gene encoding LEDGF/p75 protects cells against HIV infection
Source: Sci Rep. 2019 Feb 20;9:2389. doi: 10.1038/s41598-019-38718-0 (PMC6382798; doi:10.1038/s41598-019-38718-0)
Supplement: Supplementary file 1 — Supplemental Figures [file 41598_2019_38718_MOESM1_ESM.pptx]

## Slide 1
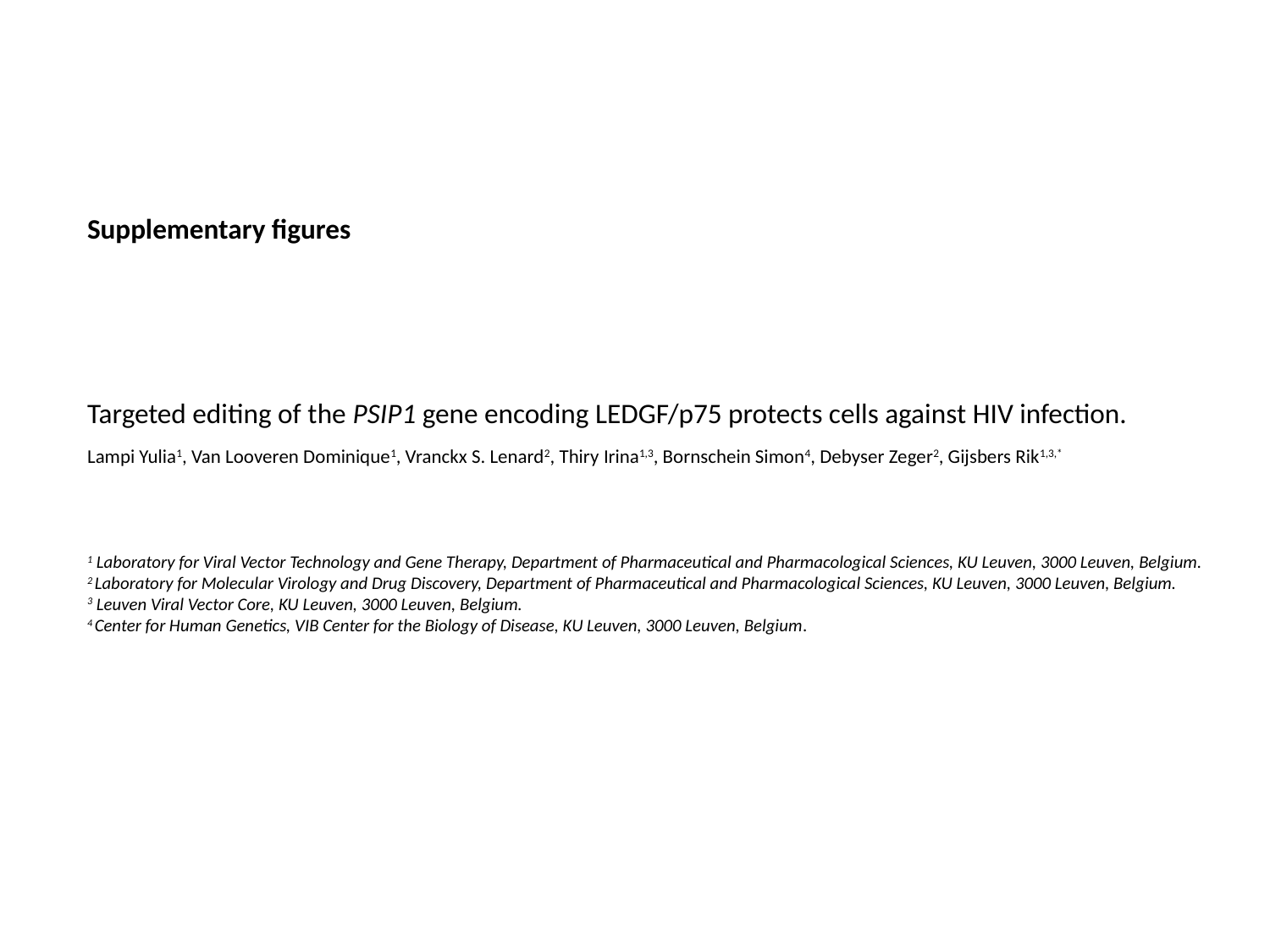

Supplementary figures
Targeted editing of the PSIP1 gene encoding LEDGF/p75 protects cells against HIV infection.
Lampi Yulia1, Van Looveren Dominique1, Vranckx S. Lenard2, Thiry Irina1,3, Bornschein Simon4, Debyser Zeger2, Gijsbers Rik1,3,*
1 Laboratory for Viral Vector Technology and Gene Therapy, Department of Pharmaceutical and Pharmacological Sciences, KU Leuven, 3000 Leuven, Belgium.
2 Laboratory for Molecular Virology and Drug Discovery, Department of Pharmaceutical and Pharmacological Sciences, KU Leuven, 3000 Leuven, Belgium.
3 Leuven Viral Vector Core, KU Leuven, 3000 Leuven, Belgium.
4 Center for Human Genetics, VIB Center for the Biology of Disease, KU Leuven, 3000 Leuven, Belgium.

## Slide 2
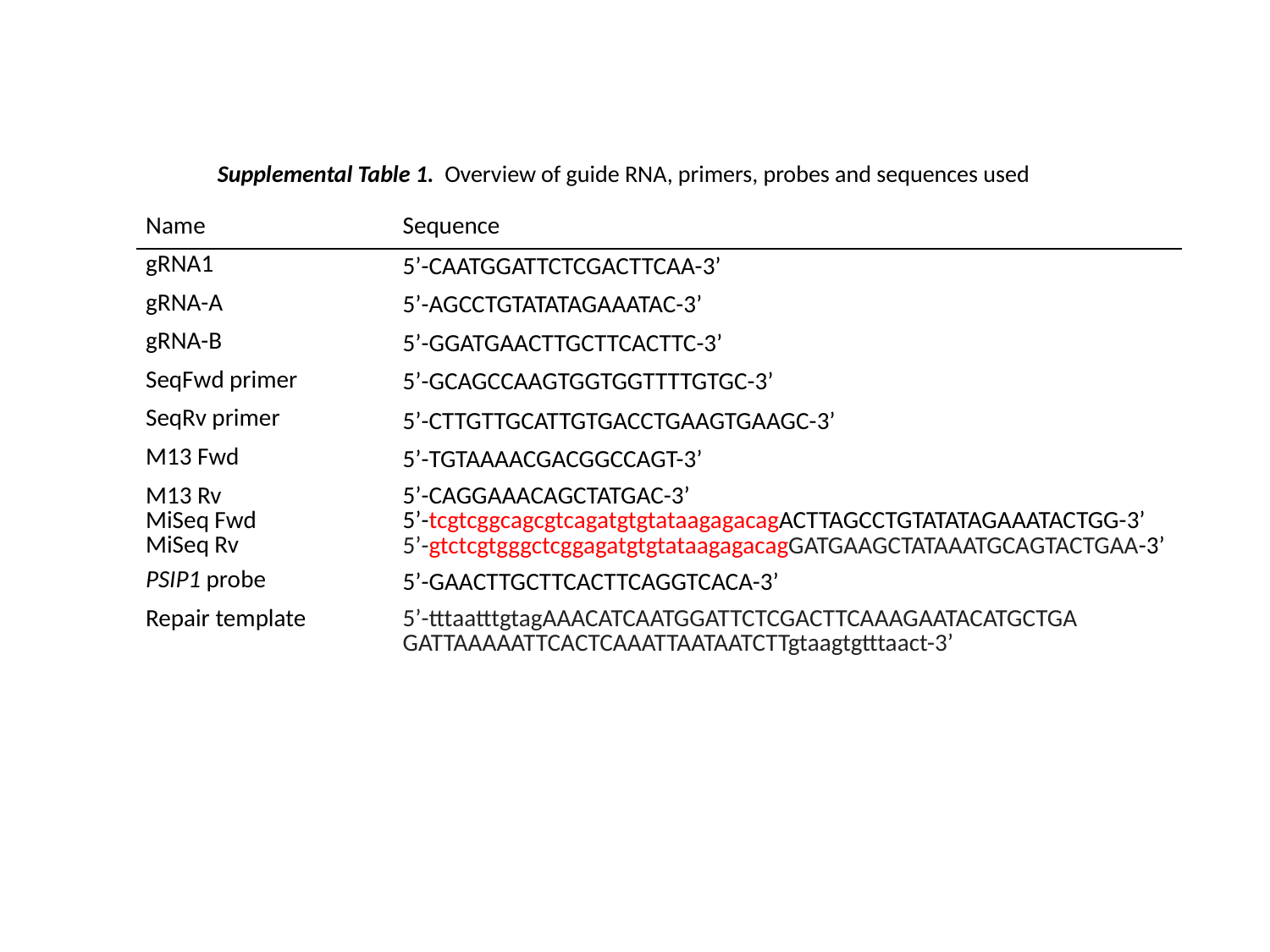

Supplemental Table 1. Overview of guide RNA, primers, probes and sequences used
| Name | Sequence |
| --- | --- |
| gRNA1 | 5’-CAATGGATTCTCGACTTCAA-3’ |
| gRNA-A | 5’-AGCCTGTATATAGAAATAC-3’ |
| gRNA-B | 5’-GGATGAACTTGCTTCACTTC-3’ |
| SeqFwd primer | 5’-GCAGCCAAGTGGTGGTTTTGTGC-3’ |
| SeqRv primer | 5’-CTTGTTGCATTGTGACCTGAAGTGAAGC-3’ |
| M13 Fwd | 5’-TGTAAAACGACGGCCAGT-3’ |
| M13 Rv MiSeq Fwd MiSeq Rv | 5’-CAGGAAACAGCTATGAC-3’  5’-tcgtcggcagcgtcagatgtgtataagagacagACTTAGCCTGTATATAGAAATACTGG-3’ 5’-gtctcgtgggctcggagatgtgtataagagacagGATGAAGCTATAAATGCAGTACTGAA-3’ |
| PSIP1 probe | 5’-GAACTTGCTTCACTTCAGGTCACA-3’ |
| Repair template | 5’-tttaatttgtagAAACATCAATGGATTCTCGACTTCAAAGAATACATGCTGA GATTAAAAATTCACTCAAATTAATAATCTTgtaagtgtttaact-3’ |

## Slide 3
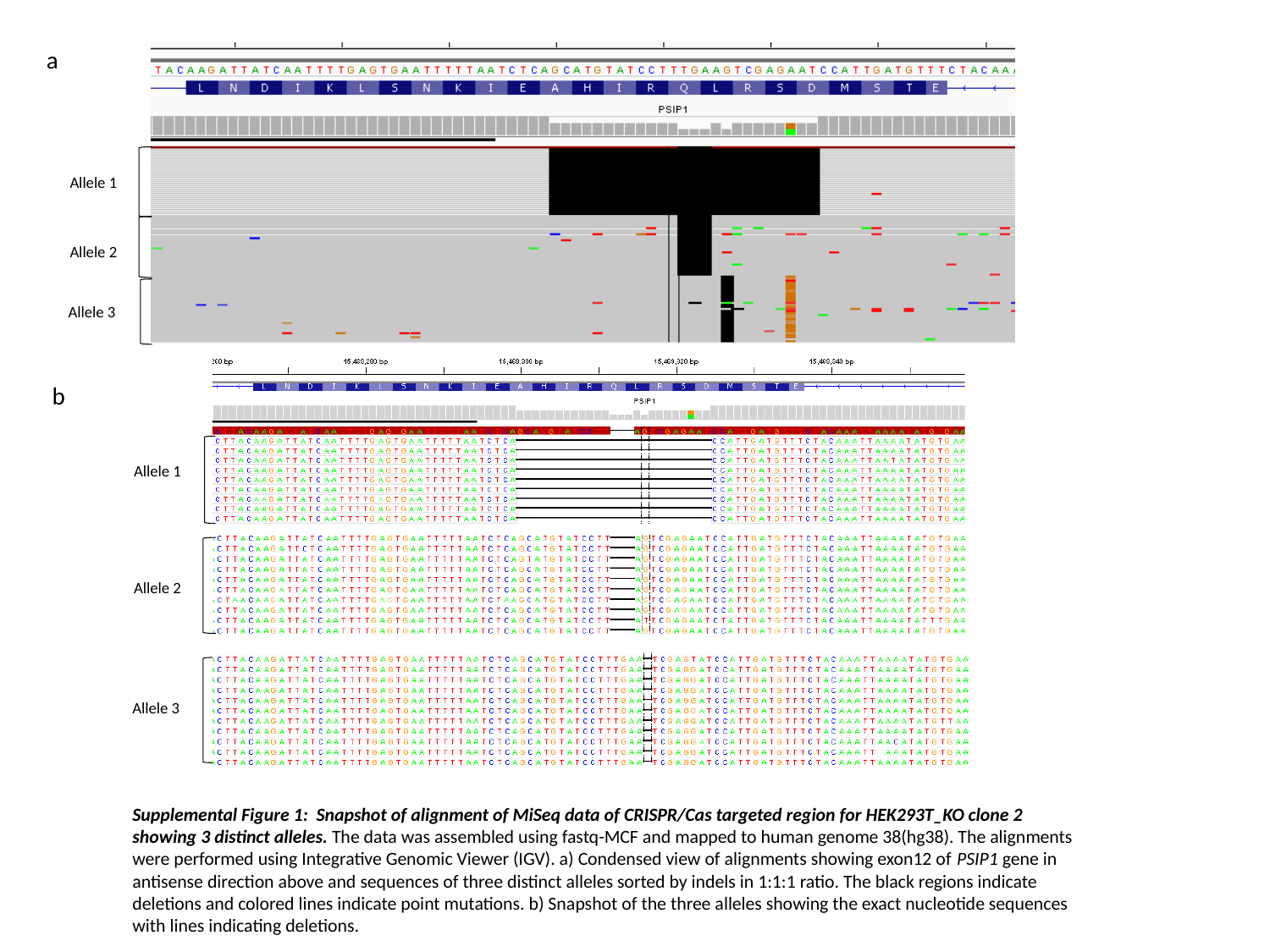

a
Allele 1
Allele 2
Allele 3
b
Allele 1
Allele 2
Allele 3
Supplemental Figure 1: Snapshot of alignment of MiSeq data of CRISPR/Cas targeted region for HEK293T_KO clone 2 showing 3 distinct alleles. The data was assembled using fastq-MCF and mapped to human genome 38(hg38). The alignments were performed using Integrative Genomic Viewer (IGV). a) Condensed view of alignments showing exon12 of PSIP1 gene in antisense direction above and sequences of three distinct alleles sorted by indels in 1:1:1 ratio. The black regions indicate deletions and colored lines indicate point mutations. b) Snapshot of the three alleles showing the exact nucleotide sequences with lines indicating deletions.

## Slide 4
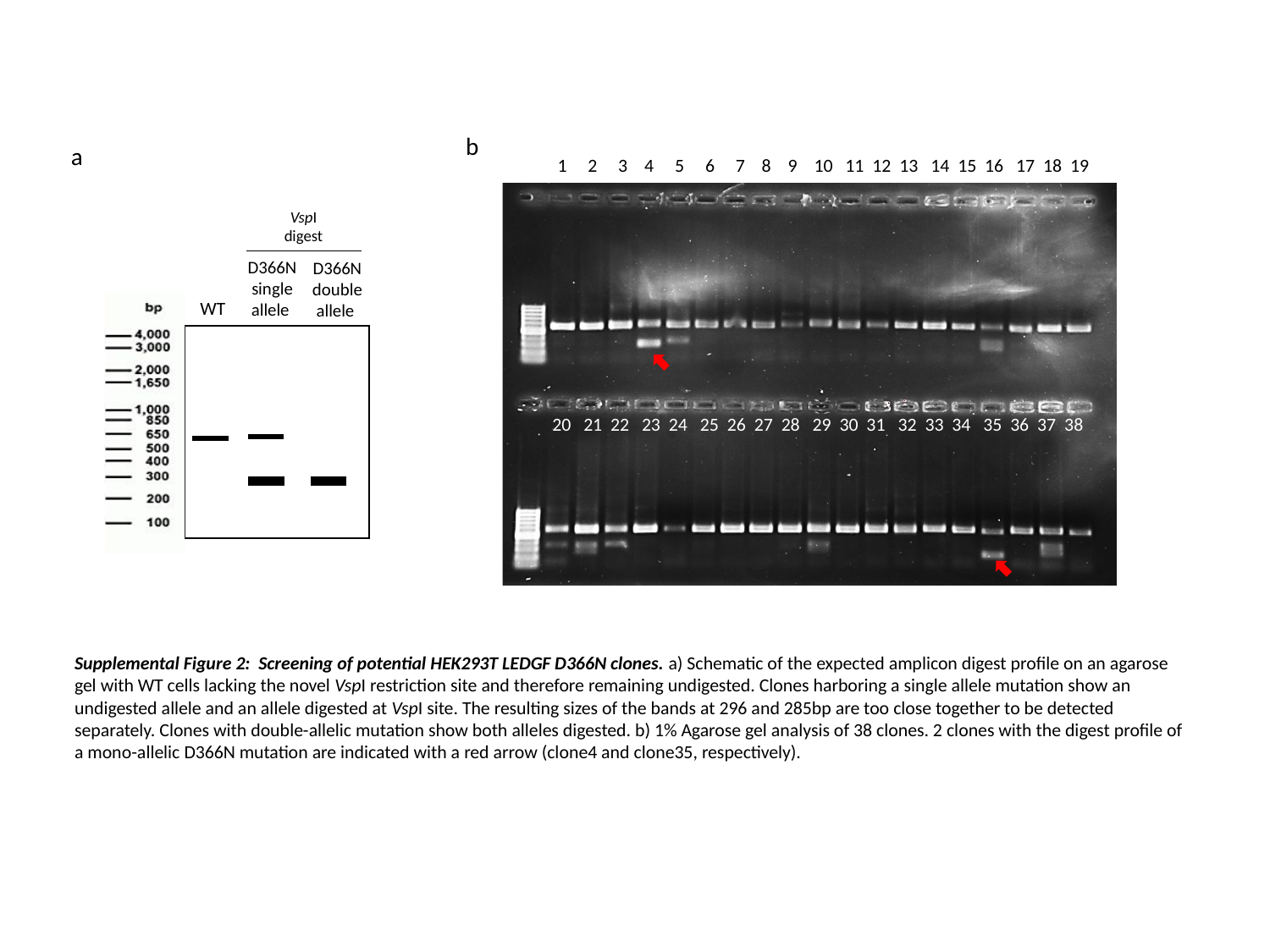

b
a
1 2 3 4 5 6 7 8 9 10 11 12 13 14 15 16 17 18 19
VspI digest
D366N single allele
D366N double allele
WT
20 21 22 23 24 25 26 27 28 29 30 31 32 33 34 35 36 37 38
Supplemental Figure 2: Screening of potential HEK293T LEDGF D366N clones. a) Schematic of the expected amplicon digest profile on an agarose gel with WT cells lacking the novel VspI restriction site and therefore remaining undigested. Clones harboring a single allele mutation show an undigested allele and an allele digested at VspI site. The resulting sizes of the bands at 296 and 285bp are too close together to be detected separately. Clones with double-allelic mutation show both alleles digested. b) 1% Agarose gel analysis of 38 clones. 2 clones with the digest profile of a mono-allelic D366N mutation are indicated with a red arrow (clone4 and clone35, respectively).

## Slide 5
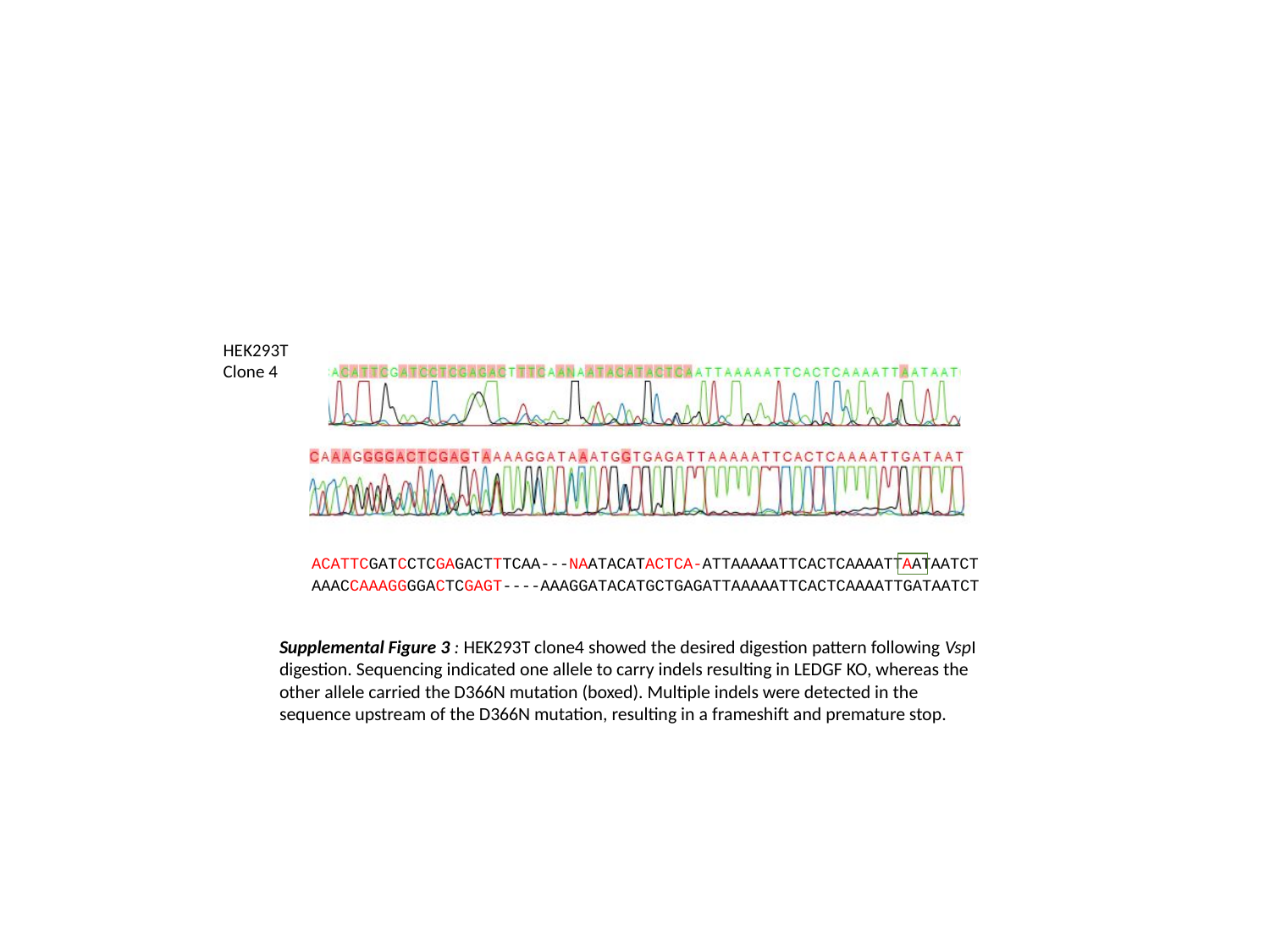

HEK293T Clone 4
ACATTCGATCCTCGAGACTTTCAA---NAATACATACTCA-ATTAAAAATTCACTCAAAATTAATAATCT
AAACCAAAGGGGACTCGAGT----AAAGGATACATGCTGAGATTAAAAATTCACTCAAAATTGATAATCT
Supplemental Figure 3 : HEK293T clone4 showed the desired digestion pattern following VspI digestion. Sequencing indicated one allele to carry indels resulting in LEDGF KO, whereas the other allele carried the D366N mutation (boxed). Multiple indels were detected in the sequence upstream of the D366N mutation, resulting in a frameshift and premature stop.

## Slide 6
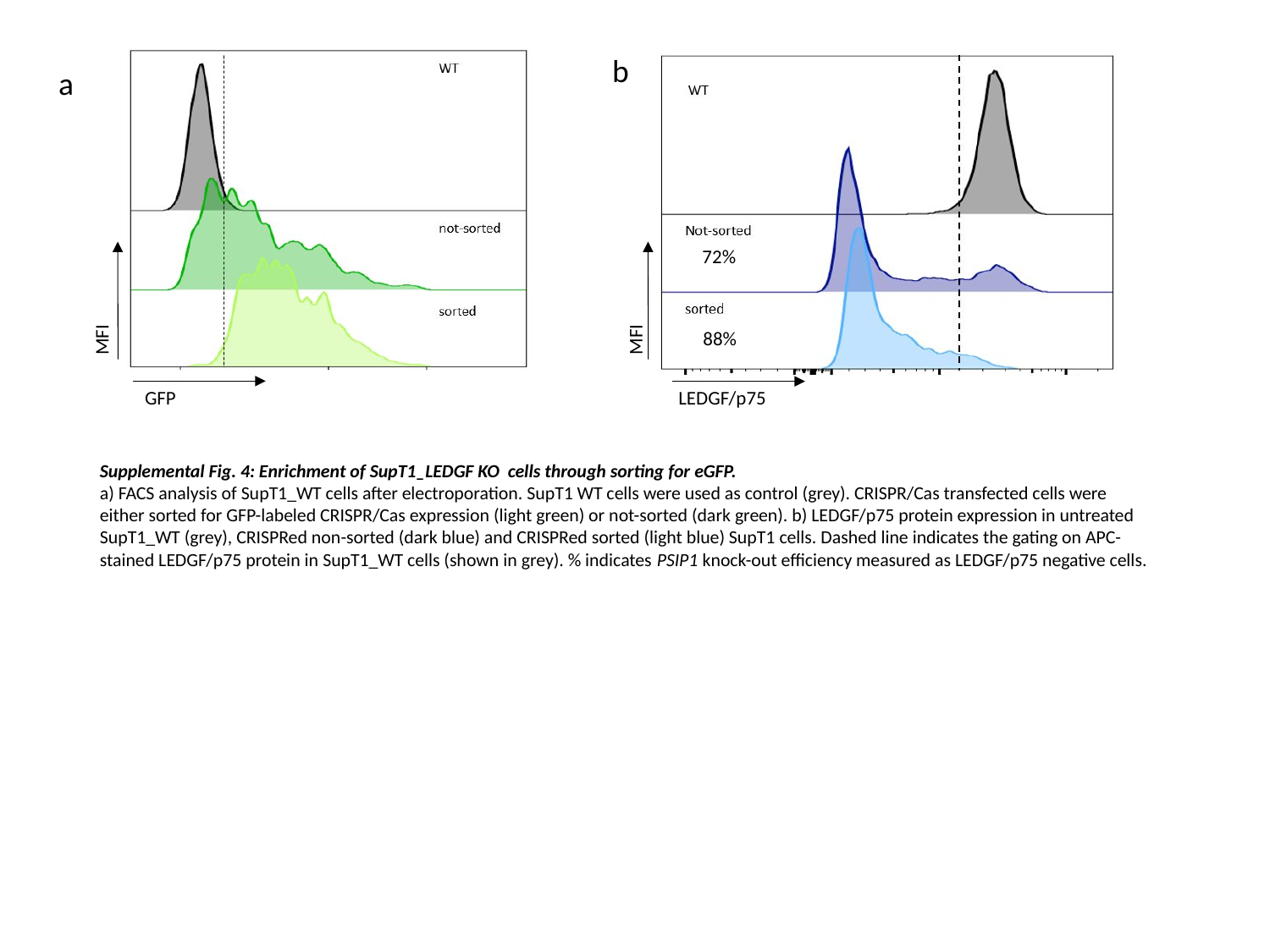

b
a
72%
MFI
MFI
88%
GFP
LEDGF/p75
Supplemental Fig. 4: Enrichment of SupT1_LEDGF KO cells through sorting for eGFP.
a) FACS analysis of SupT1_WT cells after electroporation. SupT1 WT cells were used as control (grey). CRISPR/Cas transfected cells were either sorted for GFP-labeled CRISPR/Cas expression (light green) or not-sorted (dark green). b) LEDGF/p75 protein expression in untreated SupT1_WT (grey), CRISPRed non-sorted (dark blue) and CRISPRed sorted (light blue) SupT1 cells. Dashed line indicates the gating on APC-stained LEDGF/p75 protein in SupT1_WT cells (shown in grey). % indicates PSIP1 knock-out efficiency measured as LEDGF/p75 negative cells.

## Slide 7
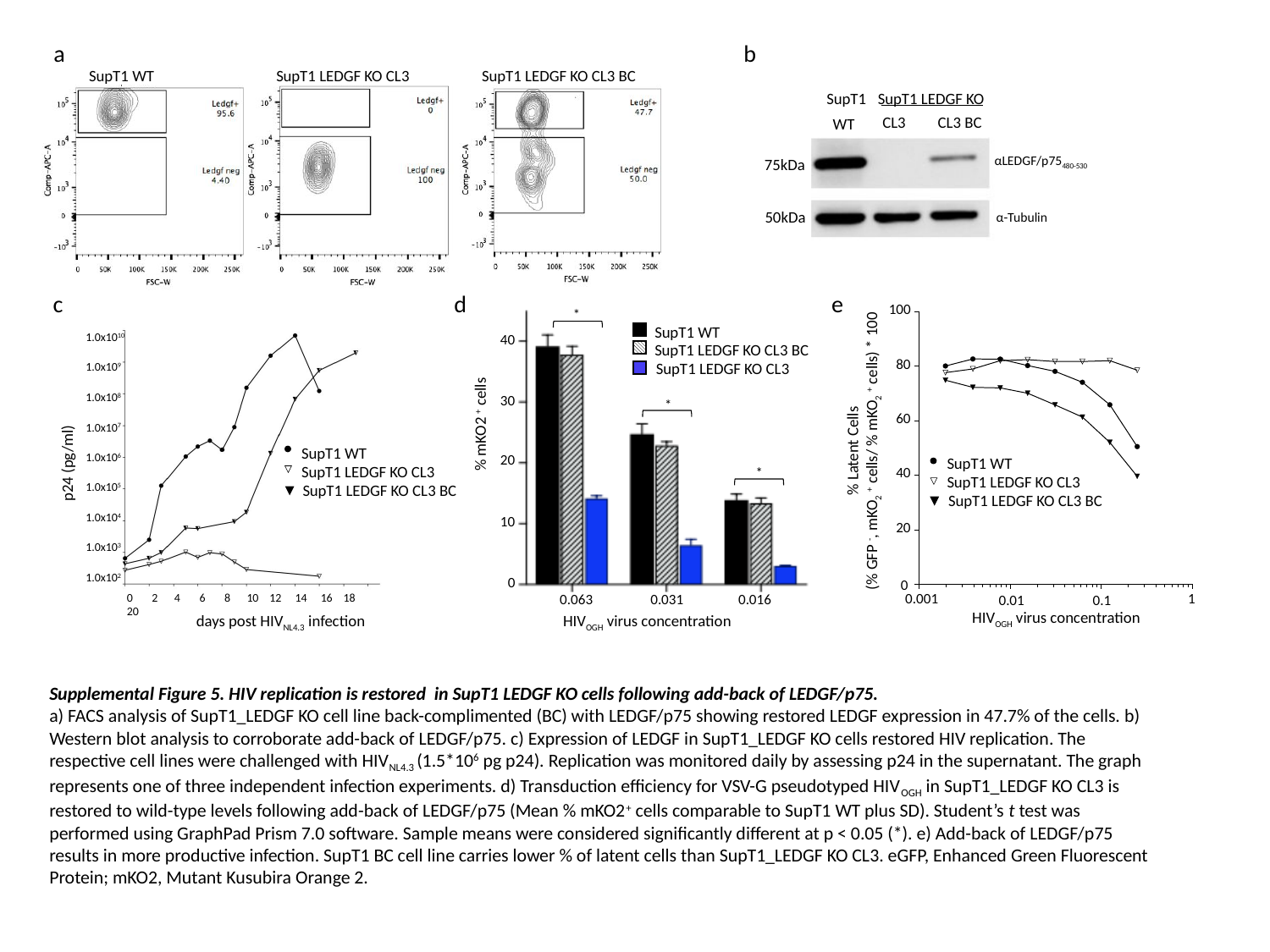

a
b
SupT1 WT
SupT1 LEDGF KO CL3
SupT1 LEDGF KO CL3 BC
SupT1 LEDGF KO
SupT1
CL3 BC
CL3
WT
 αLEDGF/p75480-530
75kDa
50kDa
α-Tubulin
c
d
e
100
*
1.0x10^10^
1.0x10^9^
1.0x10^8^
 p24 (pg/ml)
1.0x10^7^
SupT1 WT
1.0x10^6^
SupT1 LEDGF KO CL3
1.0x10^5^
SupT1 LEDGF KO CL3 BC
1.0x10^4^
1.0x10^3^
1.0x10^2^
0 2 4 6 8 10 12 14 16 18 20
SupT1 WT
40
SupT1 LEDGF KO CL3 BC
80
SupT1 LEDGF KO CL3
% mKO2 + cells
30
*
60
% Latent Cells
(% GFP -, mKO2 + cells/ % mKO2 + cells) * 100
20
SupT1 WT
SupT1 LEDGF KO CL3
SupT1 LEDGF KO CL3 BC
40
*
10
20
0
0
0.001
1
0.063
0.016
0.031
0.01
0.1
HIVOGH virus concentration
days post HIVNL4.3 infection
HIVOGH virus concentration
Supplemental Figure 5. HIV replication is restored in SupT1 LEDGF KO cells following add-back of LEDGF/p75.
a) FACS analysis of SupT1_LEDGF KO cell line back-complimented (BC) with LEDGF/p75 showing restored LEDGF expression in 47.7% of the cells. b) Western blot analysis to corroborate add-back of LEDGF/p75. c) Expression of LEDGF in SupT1_LEDGF KO cells restored HIV replication. The respective cell lines were challenged with HIVNL4.3 (1.5*106 pg p24). Replication was monitored daily by assessing p24 in the supernatant. The graph represents one of three independent infection experiments. d) Transduction efficiency for VSV-G pseudotyped HIVOGH in SupT1_LEDGF KO CL3 is restored to wild-type levels following add-back of LEDGF/p75 (Mean % mKO2+ cells comparable to SupT1 WT plus SD). Student’s t test was performed using GraphPad Prism 7.0 software. Sample means were considered significantly different at p < 0.05 (*). e) Add-back of LEDGF/p75 results in more productive infection. SupT1 BC cell line carries lower % of latent cells than SupT1_LEDGF KO CL3. eGFP, Enhanced Green Fluorescent Protein; mKO2, Mutant Kusubira Orange 2.

## Slide 8
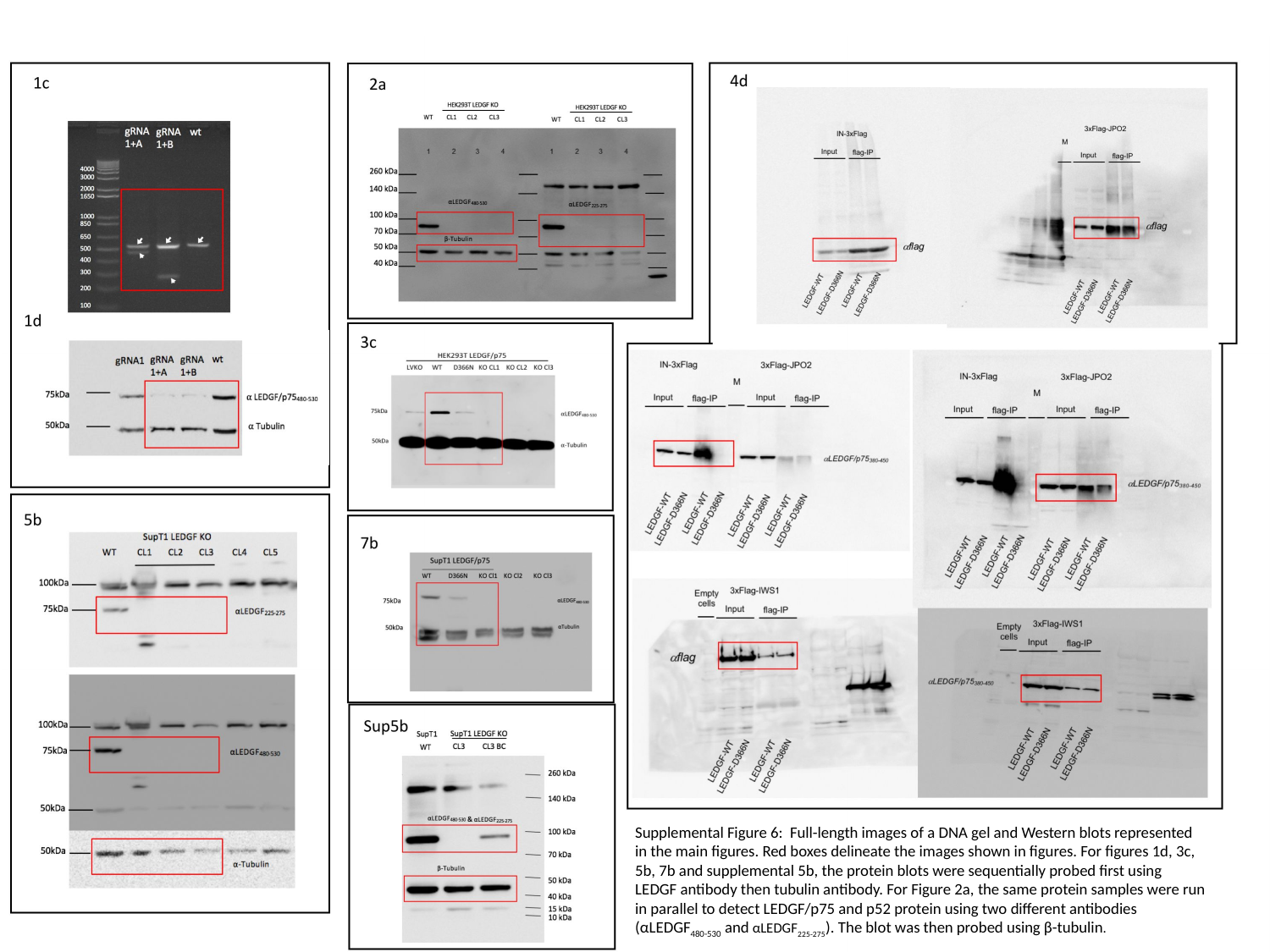

Supplemental Figure 6: Full-length images of a DNA gel and Western blots represented in the main figures. Red boxes delineate the images shown in figures. For figures 1d, 3c, 5b, 7b and supplemental 5b, the protein blots were sequentially probed first using LEDGF antibody then tubulin antibody. For Figure 2a, the same protein samples were run in parallel to detect LEDGF/p75 and p52 protein using two different antibodies (αLEDGF480-530 and αLEDGF225-275). The blot was then probed using β-tubulin.
